# Metabolic Markers and Association of Biological Sex in Lupus Nephritis

**DOI:** 10.3390/ijms242216490

**Published:** 2023-11-18

**Authors:** Bethany Wolf, Calvin R. K. Blaschke, Sandy Mungaray, Bryan T. Weselman, Mariia Stefanenko, Mykhailo Fedoriuk, Hongxia Bai, Jessalyn Rodgers, Oleg Palygin, Richard R. Drake, Tamara K. Nowling

**Affiliations:** 1 Department of Public Health Sciences, Medical University of South Carolina, 135 Cannon Street, Suite 303 MSC 835, Charleston, SC 29425, USA; wolfb@musc.edu; 2 Department of Cell and Molecular Pharmacology and Experimental Therapeutics, Medical University of South Carolina, 173 Ashley Avenue Basic Science Building 358, Charleston, SC 29425, USAbtw24@georgetown.edu (B.T.W.); baih@musc.edu (H.B.); draker@musc.edu (R.R.D.); 3 Division of Rheumatology, Department of Medicine, Medical University of South Carolina, 171 Ashley Avenue, Charleston, SC 29425, USA; mungaray@musc.edu (S.M.); ierardi@musc.edu (J.R.); 4 Division of Nephrology, Department of Medicine, Medical University of South Carolina, Clinical Sciences Building, 96 Jonathan Lucas Street, Charleston, SC 29425, USA; stefanen@musc.edu (M.S.); fedoriuk@musc.edu (M.F.); palygin@musc.edu (O.P.)

**Keywords:** glycosylation, N-glycan, glycosphingolipid, lupus nephritis, mesangial cell, sex bias, biomarker

## Abstract

Lupus nephritis (LN) is a serious complication for many patients who develop systemic lupus erythematosus, which primarily afflicts women. Our studies to identify biomarkers and the pathogenic mechanisms underlying LN will provide a better understanding of disease progression and sex bias, and lead to identification of additional potential therapeutic targets. The glycosphingolipid lactosylceramide (LacCer) and N-linked glycosylated proteins (N-glycans) were measured in urine and serum collected from LN and healthy control (HC) subjects (10 females and 10 males in each group). The sera from the LN and HC subjects were used to stimulate cytokine secretion and intracellular Ca^2+^ flux in female- and male-derived primary human renal mesangial cells (hRMCs). Significant differences were observed in the urine of LN patients compared to HCs. All major LacCers species were significantly elevated and differences between LN and HC were more pronounced in males. 72 individual N-glycans were altered in LN compared to HC and three N-glycans were significantly different between the sexes. In hRMCs, Ca^2+^ flux, but not cytokine secretion, was higher in response to LN sera compared to HC sera. Ca^2+^ flux, cytokine secretion, and glycosphingolipid levels were significantly higher in female-derived compared to male-derived hRMCs. Relative abundance of some LacCers and hexosylceramides were higher in female-derived compared to male-derived hRMCs. Urine LacCers and N-glycome could serve as definitive LN biomarkers and likely reflect renal disease activity. Despite higher sensitivity of female hRMCs, males may experience greater increases in LacCers, which may underscore worse disease in males. Elevated glycosphingolipid metabolism may poise renal cells to be more sensitive to external stimuli.

## 1. Introduction

Systemic lupus erythematosus (SLE) is an autoimmune disease in which the immune system can attack a variety of organs. Nephritis is a major complication of lupus that occurs in greater than 50% of SLE patients. SLE also exhibits a strong sex bias occurring 9–10 times more frequently in females than males [1]. The underlying mechanisms involved in the development of nephritis in SLE patients are not completely known, nor is the sex bias in disease understood. While many studies have focused on understanding changes in the levels of genes or proteins, few have investigated the changes in lipids or glycosylation with respect to disease or sex bias.

Glycosphingolipids (GSLs) are neutral lipids synthesized from ceramide by the addition of galactose or glucose to generate galactosylceramides (GalCers) or glucosylceramides (GlcCers), which together make up hexosylceramides (HexCers). Lactosylceramides (LacCers) are generated from GlcCers. These GSLs are involved in a wide array of functions in most cell types, including proliferation, apoptosis, and signal transduction [2,3,4,5,6]. We previously demonstrated in a small cohort of subjects that LacCers were significantly elevated in the urine of lupus patients with nephritis compared to lupus patients without nephritis or healthy subjects [7]. Differences in these GSLs were not observed in the serum. While sex differences of the circulating lipidome were recently reported, GSLs (GlcCers and LacCers) were not included [8]. To our knowledge, quantification of circulating or urine GSLs with respect to sex in healthy subjects, or to sex and disease in SLE patients, has not been reported.

Similar to GSLs, N-linked glycosylation of lipids and proteins plays an important role in mediating many different cellular functions including cell interactions and signal transduction. N-linked glycosylation can modulate the activity of proteins including IgG effector function [9]. While changes in the N-glycome are observed in many inflammatory or autoimmune diseases [10,11] including lupus [12,13,14,15,16,17], global changes in the N-glycome associated with disease or with biologic sex in lupus nephritis are unknown.

In this study, we analyzed differences in the levels of LacCers and N-glycosylated proteins (N-glycans) in urine and serum with respect to disease status and biologic sex. We show that all major LacCers in the urine, but only two major LacCers in the serum, were significantly elevated in LN patients compared to HC subjects in this study cohort. Although no differences were observed in overall levels of urine or serum LacCers with respect to biologic sex, a greater increase in urine LacCers was observed in males when comparing LN to HC. We observed that 75% of the urine N-glycans and 30% of the serum N-glycans were associated with disease status. Three of the urine N-glycans were associated with biologic sex. Activation of primary human renal mesangial cells (hRMCs) to the human sera was observed by measuring intracellular Ca^2+^ flux and cytokine release. Ca^2+^ flux was significantly higher in response to serum from LN patients compared to HC subjects. Intracellular Ca^2+^ flux in hRMCs derived from a female donor was more sensitive and the cells released two-fold to ten-fold higher levels of cytokines in response to sera compared to hRMCs derived from a male donor. Interestingly, the GSLs levels were higher in the female-derived hRMCs compared to the male-derived hRMCs and this may contribute in part to the hyper-response of the female-derived hRMCs.

## 2. Results

### 2.1. Comparison of LacCers and N-Linked Glycosylation in Urine of Lupus Nephritis Patients Compared to Healthy Controls and between Sexes

**Figure 1:**
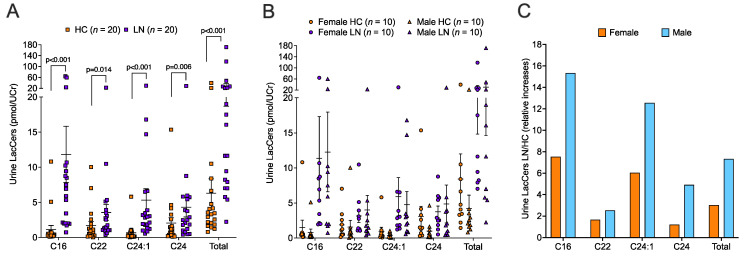
**Urine LacCers levels were significantly higher in LN compared to HC.** LacCer chain lengths of C14 to C26 were quantified in urine samples of healthy controls (HC) and lupus nephritis patients (LN) and included 10 females and 10 males in each group. (**A**) Levels of the major LacCer chain lengths detected in the urine and the total of all LacCers chain lengths (Total) are shown in the graphs for all HC (*n* = 20) compared to all LN (*n* = 20) subjects. (**B**) Levels of the major LacCer chain lengths by sex in the HC and LN groups. (**C**) Ratio of LacCers levels in LN to HC by sex.

The study population comparing lupus nephritis (LN) patients to healthy controls (HCs) included 20 subjects in each group with equal sex distribution (50% female for each group). LN patients and HCs were similar in age but a larger proportion of LN patients were black relative to HCs. The eGFR and urine creatinine levels were similar in the two groups. Significant differences between LN patients and HCs were observed for UPCr, C3 complement, and C4 complement. Participant demographics by disease status are shown in Table 1. We previously demonstrated that levels of LacCers in the urine of LN patients were significantly higher compared to lupus patients without nephritis and compared to HCs [7]. Similarly, in this study cohort, the levels of the major chain lengths of LacCer (C16, C22, C24:1, and C24), as well as the total of all LacCer chain lengths, were significantly higher in LN patients compared to HCs (Figure 1A). To determine if LacCers levels differed based on biologic sex, we compared LacCers levels in the two groups. Although the differences did not reach statistical significance in this small cohort, the urine LacCers levels tended to be higher in females compared to males in the HC group (Figure 1B). This trend was not observed in the LN group. Thus, the relative increases in LN urine LacCers (C16, C24:1, C24, and total) compared to HC urine was approximately twofold higher in LN males than in LN females (Figure 1C). This suggests that while both sexes with LN experience increases in urine LacCers, males may have a larger increase than females.

**Figure 2:**
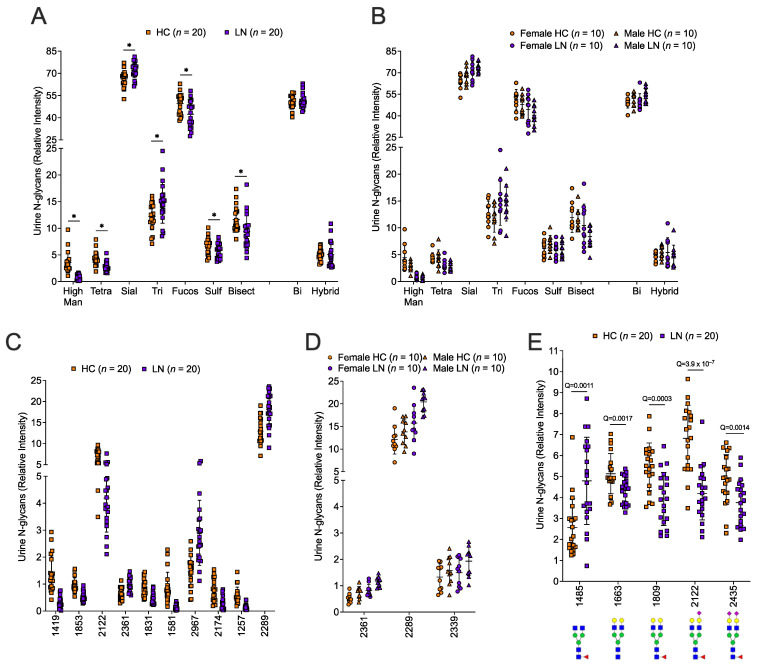
**The urine N-glycome was significantly different in LN compared to HC; some differed by sex.** N-glycans were quantified in the same urine samples as in Figure 1 of 20 healthy controls (HC) and 20 lupus nephritis patients (LN) and included 10 females and 10 males in each group. N-glycan classes: high mannose (High Man), tetra-antennary (Tetra), sialylated (Sial), tri-antennary (Tri), fucosylated (Fucos), sulfated (Sulf), bisected (Bisect), bi-antennary (Bi), or Hybrid were compared in all HC vs. all LN (**A**) or by sex in each group (**B**). * Significant difference between HC and LN. Specific Q-values are provided in Table S1. Individual N-glycans were compared in all HC vs. all LN (**C**) or by sex in each group (**D**). Of the 72 individual N-glycans that differed significantly between HC and LN, the top 10 are presented in (**C**). Only three N-glycans differed significantly between females and males (**D**). (**E**) N-glycans associated with IgG are significantly different between HC and LN. N-glycan structures shown below the m/z peak values. See Table S2 for a list of all urine N-glycans detected and the adjusted Q values and Table S1 for N-glycans structure information.

Ninety-six individual N-glycans (peaks) in urine were detectable in most of the samples, summarized in Supplemental Table S2. Nine classes of glycans, which used the sum of the relative frequencies for those peaks in that class, were also considered: mannose, hybrid, biantennary, triantennary, tetrantennary, bisecting, fucosylated, sialylated, and sulfonated. Seven of the N-glycan classes differed significantly between LN patients and HCs after FDR correction (Figure 2A). Of the 96 individual N-glycans detected, the relative abundance of 72 of the N-glycans differed significantly between LN patients and HCs after FDR correction. The top 10 significantly different individual N-glycans are presented in Figure 2C. We also examined sex differences in the relative frequencies of the glycans in these data. The interaction between biologic sex and disease status was not significant in any of the statistical models. Thus, results for disease status are reported across males and females and results for biologic sex are reported across disease status. None of the N-glycan classes differed significantly by sex (Figure 2B) and only three of the individual N-glycans (peaks 2361, 2339, and 2289, Figure 2D) differed significantly by sex after FDR correction. There was a significant increase in the degalactosylated (and desialylated, peak 1485) glycan shown to be associated with IgG that exhibits a more pro-inflammatory function, and significant decreases in the biantennary galactosylated (peaks 1663 and 1809) and sialylated (peaks 2122 and 2435) glycans that are associated with a more anti-inflammatory IgG (Figure 2E). Supplemental Figure S1A shows a heatmap of the 72 glycans found to be associated with disease status and Supplementary Table S2 shows the mean difference and 95% confidence interval in the relative frequencies of all the N-glycan classes and individual peaks between LN patients and HCs as well as between males and females.

We then examined if inclusion of the individual N-glycans in a model that included total urine LacCers level and biologic sex improved discrimination between LN patients and HCs for those N-glycans identified as differing between LN and HC. Models with one N-glycan added were compared to the baseline model for improvements in fit based on the likelihood ratio test. Of the 72 N-glycans identified to be associated with disease, 27 were significant in a model including total urine LacCers and biologic sex (although none retained significance after FDR correction). The primary metric for evaluating discrimination of cases was the AUC statistic. The baseline model including only urine total LacCers and biologic sex had an AUC (95% CI) of 0.87 (0.75, 0.99). Models including one additional N-glycan from among those associated with SLE status improved the AUC relative to the baseline model by between 0 and 0.118 units (i.e., increasing the AUC from 0.870 to between 0.870–0.992). The AUCs, likelihood ratio test *p*-values (for comparison of a model including the N-glycan versus excluding the N-glycan), and difference in AUCs are shown in Table 2.

### 2.2. Comparison of LacCers and N-Linked Glycosylation in Serum of Lupus Nephritis Patients Compared to Healthy Controls and between Sexes

**Figure 3:**
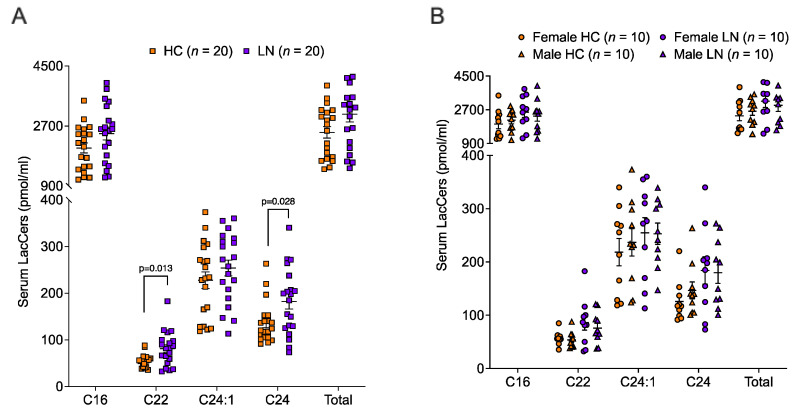
**Two serum LacCer species were significantly higher in serum from LN compared to HC.** LacCer chain lengths of C14 to C26 were quantified in serum samples of the same HC and LN subjects as in Figure 1 (10 females and 10 males in each group). (**A**) Levels of the major LacCer chain lengths detected in the serum and the total of all LacCers chain lengths (Total) are shown in the graphs for all HC (*n* = 20) compared to all LN (*n* = 20) subjects. (**B**) Levels of the major LacCer chain lengths by sex in the HC and LN groups.

Serum samples were analyzed from the same LN patients and HCs from whom urine samples were analyzed above. As we reported previously in a different cohort of subjects [7], we did not observe a significant difference in C16 LacCer levels between LN patients and HCs in this cohort, nor did we observe a difference in the levels of total LacCers (Figure 3A). However, we did observe significant differences in C22 and C24 LacCers between LN patients and HCs. As in the urine LacCers analyses, we did not observe any differences in serum LacCers based on biological sex regardless of disease status (Figure 3B).

**Figure 4:**
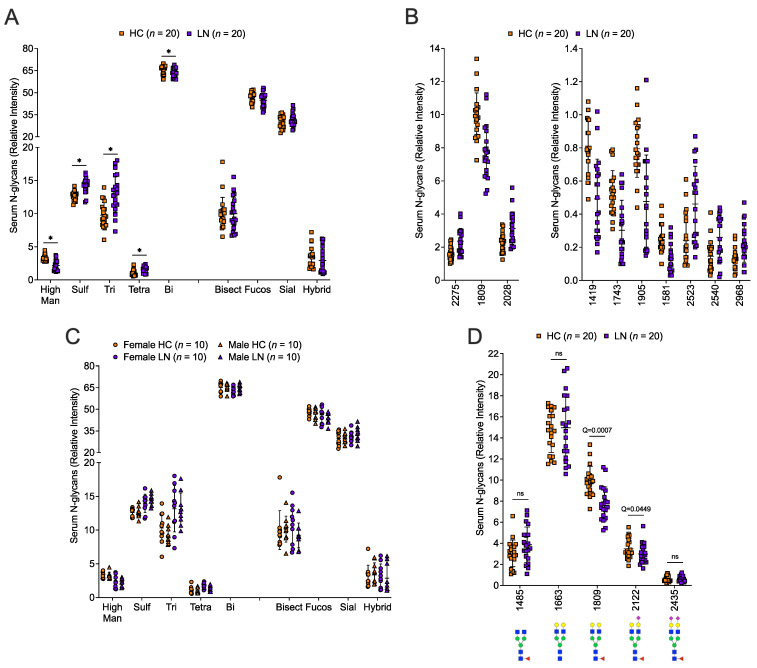
**The serum N-glycome was significantly different in LN compared to HC.** N-glycans were quantified in the same serum samples as in Figure 3 of 20 healthy controls (HC) and 20 lupus nephritis patients (LN) and included 10 females and 10 males in each group. N-glycan classes: high mannose (High Man), tetra-antennary (Tetra), sialylated (Sial), tri-antennary (Tri), fucosylated (Fucos), sulfated (Sulf), bisected (Bisect), bi-antennary (Bi), or Hybrid were compared in all HC vs. all LN (**A**) or by sex in each group (**C**) * Q < 0.05 (see Table S3 for calculated adjusted Q-values). (**B**) Individual N-glycans were compared in all HC vs. all LN and the top 10 of the 21 individual N-glycans that differed significantly between HC and LN are presented. (**D**) N-glycans associated with IgG (ns, not significant).. N-glycan structures shown below the m/z peak values. See Table S3 for a list of all serum N-glycans detected and the calculated adjusted Q-values and Table S1 for N-glycans structures.

Seventy individual N-glycans (peaks) were detectable in serum in most of the samples. Five N-glycan classes differed significantly between LN patients and HCs after FDR correction (Figure 4A). Of the 70 detected individual N-glycans, the relative abundance of 21 of the N-glycans groups differed significantly between LN patients and HCs after FDR correction. The top 10 significantly different individual N-glycans are shown in Figure 4B. We also examined sex differences in the relative frequencies of the N-glycans in these data; however, none of the N-glycan classes (Figure 4C) or individual N-glycans differed significantly by sex after FDR correction. Only one of the IgG-associated N-glycan peaks, 1809 (desialylated containing a core fucose), in the serum was highly significantly different between the two groups with it being decreased in the LN group (Figure 4D). The mono-sialylated form was also decreased with the difference only just significant at Q = 0.0449. Supplementary Figure S1B shows a heatmap of the 26 serum N-glycans (21 individual and 5 classes) associated with disease status. Supplementary Table S3 shows the mean difference and 95% confidence interval in the relative frequencies of the different N-glycan classes or individual N-glycans between LN patients and HCs, and between males and females.

We then examined if inclusion of the individual serum N-glycans in a model including total urine LacCers level and biologic sex improved discrimination between LN and HC for those N-glycans identified as differing between LN patients and HCs. Models with one N-glycan added were compared to the baseline model for improvements in fit base on the likelihood ratio test. Of the 21 individual or 5 classes of N-glycans associated with SLE status, 20 were significant in a model including total urine LacCers and biologic sex (although none retained significance after FDR correction). The baseline model including only urine total LacCers and biologic sex had an AUC (95% CI) of 0.87 (0.75, 0.99). Models including one additional N-glycan from among those identified to be associated with LN status improved the AUC relative to the baseline model by between 0.005 and 0.085 units. The AUCs, likelihood ratio test *p*-values (for comparison of a model including the serum N-glycan versus excluding the N-glycan), and difference in AUCs are shown in Table 3.

### 2.3. Influence of Disease and Biologic Sex in the Response of Mesangial Cells to Human Sera

The above results showed significant differences in the levels of 21 different N-glycans between LN and HC serum. To determine if renal cells would exhibit differential responses to these sera, primary human renal mesangial cells (hRMCs) were used for the following studies. A preliminary experiment was performed to identify cytokines that may be differentially secreted in response to LN compared to HC sera (see Supplemental methods). We first measured the release of IL-6 and MCP-1 in response to 10% sera collected from 12 HCs, 12 LN patients with active disease, and 12 LN patients with inactive disease (not the same subjects included the LacCers and N-glycan analyses) based on our prior studies in mice. [18,19]. No differences across the three groups were observed in IL-6 or MCP-1 release (Figure S2A). The media from this experiment was then pooled to generate two samples per group and a cytokine array screened to identify cytokines that may be differentially released in response to HC vs. LN Active vs. LN Inactive sera. Results from this array (Figure S2B) suggested that higher levels of CCL5 and CXCL5 were released in response to LN Active sera compared to LN Inactive or HC sera.

**Figure 5:**
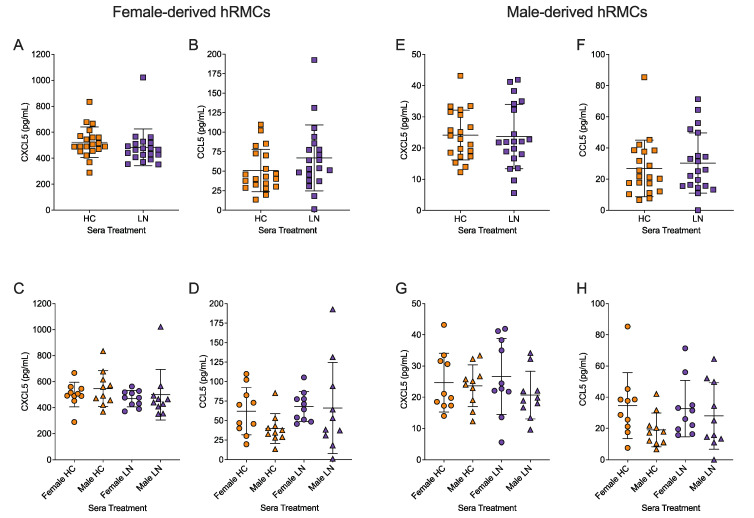
**Cytokine release was higher in female-derived compared to male-derived hRMCs in response to sera.** hRMCs were treated with 5% serum from 10 female or 10 male HC or from 10 female or 10 male LN subjects. (**A**–**D**): female-derived hRMCs; and (**E**–**H**): male-derived hRMCs. (**A**,**C**,**E**,**G**): CXCL5; and (**B**,**D**,**F**,**H**): CCL5 levels in media of sera-treated hRMCs. (**A**,**B**,**E**,**F**): compares HC sera-treated to LN sera-treated (both female- and male-derived sera). (**C**,**D**,**G**,**H**): compares female- and male-derived sera for each group (HC vs. LN). Statistical analyses were performed using unpaired t-tests for comparing HC vs. LN or one-way Anova for comparing female vs. male across the two groups. No significant differences were observed after correcting for multiple comparisons.

To evaluate hRMC response to the sera from the HC subjects and LN patients analyzed in Figure 3 and Figure 4, release of CCL5 and CXCL5 was measured after incubation with 5% serum from each subject. For these studies, we also examined if responses were impacted by the biologic sex from which the hRMCs were derived. No significant differences were observed between HC and LN sera treatments in the levels of CXCL5 (Figure 5A) or CCL5 (Figure 5B) released from the female-derived hRMCs. No significant differences in the release of CXCL5 (Figure 5C) were present in response to sera with respect to biologic sex. However, a trend towards higher levels of CCL5 released in response to female HC compared to male HC sera was observed but not in response to female LN vs. male LN sera (Figure 5D). Similar results were obtained in the male-derived hRMCs (Figure 5E–H), including a trend towards release of higher levels of CCL5 in response to female HC vs. male HC sera (Figure 5G). Interestingly, we observed that the levels of CXCL5 and CCL5 released were ~twenty-fold and ~two-fold higher, respectively, from the female-derived hRMCs (Figure 5A–C) compared to the male-derived hRMCs (Figure 5E–H). These differences were significant (*p* < 0.001 for both CXCL5 and CCL5). These results suggest that the female-derived cells are pre-disposed to be hyper responsive to stimuli compared to the male-derived cells.

**Figure 6:**
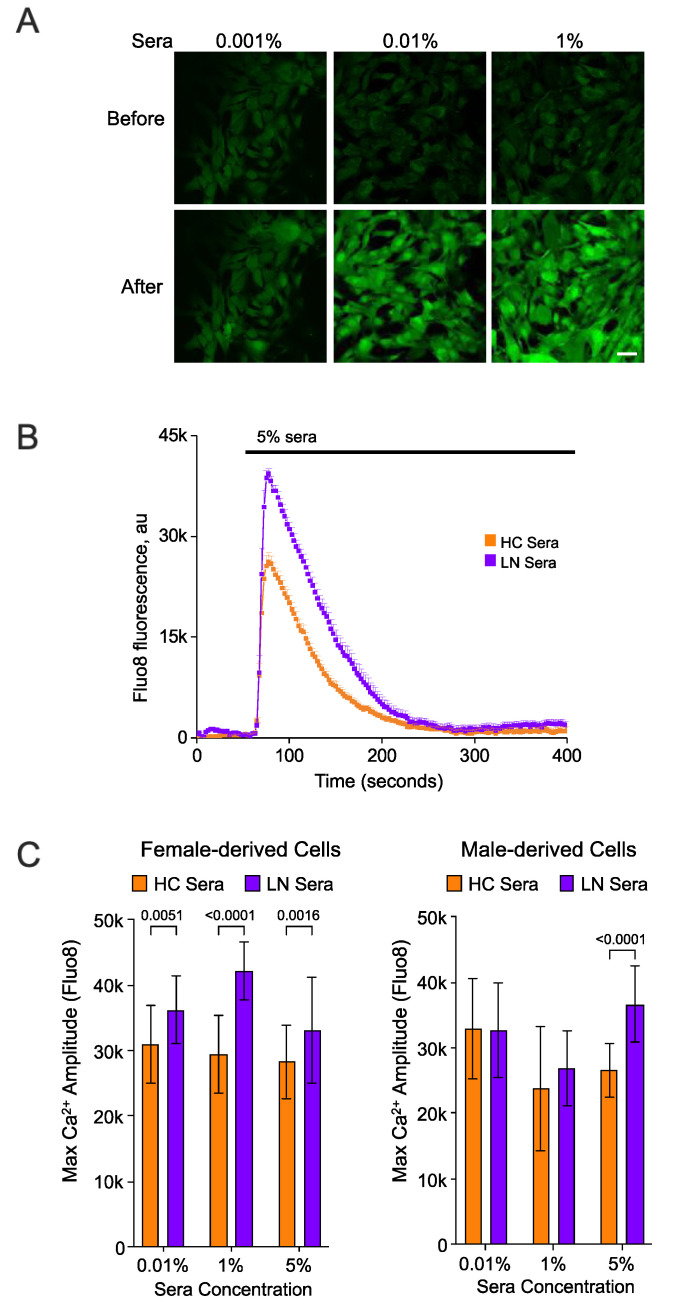
**LN sera elicited a higher intracellular Ca^2+^ flux compared to HC sera in hRMCs.** Sera was pooled from 10 female or 10 male HC (Control) or from 10 female or 10 male LN subjects and used to stimulate human primary hRMCs. Female-derived hRMCs were treated with HC or LN sera from females. Male-derived hRMCs were treated with HC or LN sera from males. (**A**) Confocal fluorescent images of intracellular Ca^2+^ (Fluo-8 AM) in female hRMCs before and after addition of 0.01%, 0.01%, or 1% female HC sera. Scale bar 50 µm. (**B**) Example of intracellular Ca^2+^ flux in response to acute application of 5% LN or HC sera in male hRMCs. (**C**) Statistical analyses of maximal amplitude of intracellular Ca^2+^ flux in response to acute sera application.

To further assess the response of hRMCs to LN versus HC sera and the effect of biological sex of the cells, we measured intracellular calcium [Ca^2+^]_i_ flux in response to acute sera applications. Calcium transients in female- or male-derived hRMCs were observed in response to pooled same-sex HC or LN serum. The transient Ca^2+^ response was detected in the range of serum concentrations of 0.001%, 0.01%, 0.1%, 1%, or 5% in 2 mM Ca^2+^ extracellular solution. The lowest concentration of sera 0.001% promotes Ca^2+^ transients with the amplitude around 20% from the saturated values reached at the concentration of 0.01% sera for all groups. Figure 6A illustrates representative confocal fluorescent (Fluo 8 AM) images of intracellular Ca^2+^ levels before and after acute application of 0.001%, 0.01%, or 1% sera. Examples of intracellular Ca^2+^ flux in response to acute application of 5% LN or HC sera in male hRMCs are shown in Figure 6B. Female-derived hRMCs exhibited significantly higher intracellular Ca^2+^ flux in response to LN compared to HC sera in all range of concentrations (Figure 6C), and male-derived cells showed a similar pattern only at the highest tested sera concentrations (5%). This data together with the results presented in Figure 5 suggest that the female-derived hRMCs are more sensitive and respond more robustly to serum stimulation than the male-derived hRMCs.

### 2.4. Differences in LacCers and HexCers Levels and the N-Glycome in Female-Derived and Male-Derived hRMCs

**Figure 7:**
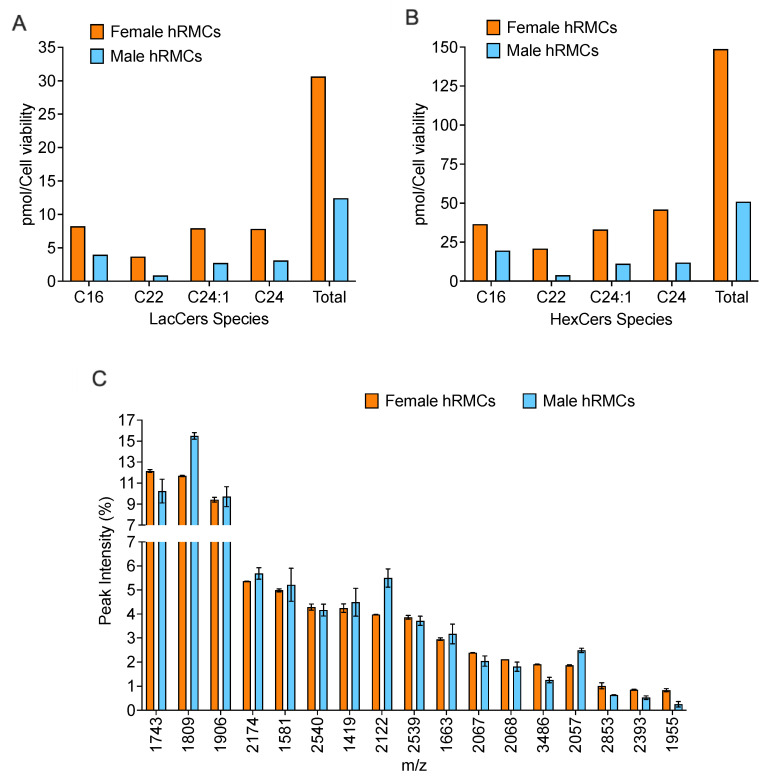
**GSL levels were higher in female-derived compared to male-derived hRMCs.** LacCers (**A**) and HexCers (**B**) were measured in female-derived and male-derived hRMCs and normalized to cell viability. The major chain lengths expressed in the hRMCs and the total of all chain lengths are shown in the graphs. The vehicle-treated female- or male-derived cells from the respective experiments presented in Figure 5 were scraped and combined to measure GLSs. GlcCers and LacCers levels were normalized to the cell viability determined prior to scraping. (**C**) N-glycans were measured by MALDI-FTICR in female- or male-derived hRMCs plated in duplicate. Individual glycans are presented as the relative peak intensity of the total glycans measured in the individual wells. Means + SD shown on graphs. Statistical analyses were not performed for the GSL or glycan analyses since the measures were performed in hRMCs from only one female donor and one male donor.

We reported previously that increasing LacCers along with another glycosphingolipid, glucosylceramides (GlcCers), resulted in increased message levels of several cytokines in an immortalized mouse mesangial cell line [19]. We also showed that LacCers and GlcCers (or hexosylceramides, a combination of GlcCers and galactosylceramides) are increased in the renal cortex of lupus prone mice with nephritis [7], and that lupus patients with nephritis that did not respond to therapy had significantly higher levels of LacCers and HexCers prior to beginning treatment [20]. Thus, we measured LacCers and hexosylceramides (HexCers) in our female- and male-derived hRMCs. The levels of both LacCers (Figure 7A) and HexCers (Figure 7B) are higher in the female-derived compared to the male-derived hRMCs prior to any stimulation. The GSLs levels in the cells in Figure 7 were measured in the serum-starved vehicle-treated wells from the experiments in Figure 5. The observed differences in GlcCers and LacCers between the female- and male-derived hRMCs were verified in unmanipulated cells maintained in serum-containing medium at passages 5 and 6 (Figure S3).

We also investigated differences in the N-glycome between the female-derived and male-derived hRMCs. The 17 most abundant N-glycans detected in both the female- and male-derived hRMCs are shown in Figure 7C. The ten most abundant N-glycans comprised >60% of all N-glycans detected in these cells. In comparing the female- and male-derived cells, the type of N-glycans present and relative overall abundance of each of the N-glycans were similar. While several glycans showed trends of being more highly abundant (1743, 3486, 2853, 2393, and 1995) or less abundant (1809, 2122, and 2057) in the female-derived cells relative to the male-derived hRMCs, these differences were not as large as those observed for the GSLs in Figure 7A,B. Thus, the analyses of GSLs and N-glycans in the hRMCs suggest that higher levels of LacCers and HexCers may contribute to a more robust response (higher cytokine release and increased intracellular Ca^2+^ flux) by the female-derived hRMCs following stimulation with sera.

## 3. Discussion

Given the ~9:1 female:male sex bias in lupus, most studies have focused largely on biologically female subjects (human and mouse studies). While men develop lupus less often than females, men were shown to have more severe disease and a higher risk of progressing to end stage renal disease [21,22]. However, the pathophysiologic mechanisms underlying sex differences are not fully understood. In this study, we determined that the significantly elevated LacCers and altered N-glycome in the urine can discriminate LN patients from HC subjects and could serve as noninvasive definitive markers of LN. Alterations in the serum N-glycome may also be useful in discriminating LN from HC, but ultimately is less informative than the urine N-glycome in this respect. While we observed a few differences in the urine N-glycome in females compared to males, the levels of urine LacCers may be more informative with respect to sex differences. A recent study reported that men with SLE develop disease at a later age [23]. Our results suggest that LN males may experience a greater increase in LacCers than females when comparing the change in LacCers levels from HC to LN. LacCers levels measured in the urine likely are derived from the kidney [24]. Therefore, we hypothesize that lower levels of LacCers may be protective in males and contribute to the later disease onset, but once tolerance is broken and males begin to develop LN, the large increase in LacCers (or possibly glycosphingolipid metabolism in general) may contribute to worse disease.

This hypothesis is supported by our results in primary human mesangial cells (hRMCs) in which the response to stimuli seems to be more dependent on cellular differences rather than on the source of circulating stimuli. hRMCs released significant levels of CXCL5 and CCL5 and exhibited significant increases in intracellular Ca^2+^ in response to human sera. The female-derived hRMCs, which we demonstrated expressed higher levels of the glycosphingolipids (GSLs) LacCers and HexCers, also released significantly more CCL5 and CXCL5 compared to the male-derived hRMCs in response to human serum (regardless of the source). At the lower concentrations of LN or HC sera, the female-derived cells had a higher intracellular Ca^2+^ flux indicating that the female-derived hRMCs have an increased sensitivity to serum stimulation. GSLs modulate cellular functions such as proliferation, apoptosis, migration, and signaling, including Ca^2+^ signaling [6], and defects in GSL metabolism are associated with a variety of human diseases. GSLs expressed on the cell surface form clusters and are widely believed to play roles in the formation and stabilization of lipid domains (“lipid rafts”) required to propagate extracellular signals. LacCers were shown to play a role in Lyn-mediated signaling in neutrophils [25,26,27] and MAPK signaling in cardiomyocytes [28], which leads to superoxide production, phagocytosis, migration, or hypertrophy. In mesangial cells, elevated LacCers and HexCers due to hyperglycemia resulted in hypertrophy, extracellular matrix production, and fibrosis [29]. Our previous studies demonstrated that LN patients that failed to respond to treatment had significantly higher levels of HexCers and LacCers prior to beginning treatment [20]. Together, these observations suggest that renal GSL metabolism plays an important role in the scope (i.e., sensitivity or magnitude) of the initial renal response to stimuli and possibly resistance to therapeutic intervention. Thus, the elevated levels of LacCers and HexCers may poise the female-derived hRMCs to respond more robustly to external stimuli than the male-derived hRMCs. We speculate that altered GSL levels or ratios in the membranes of the hRMCs may contribute in part to the increased response by the female-derived cells since these lipids play important roles in cell signaling. The increased levels in the female-derived cells may be due to differences in expression of the enzymes that modulate GSL metabolism that are regulated in part by ERα as shown in MCF cell lines [30]. Future studies designed to interrogate the expression of GSL metabolic enzymes are needed to address this question.

Post-translational N-linked glycosylation plays an important role in the function of proteins, impacting a variety of cellular functions including discriminating self from non-self. Similar to GSLs, they can play key roles in mediating cell function. Modifications of N-glycosylation, or an altered N-glycome, is associated with several human diseases including lupus [15,31,32,33]. A recent study showed an abnormal N-glycome in renal biopsy sections of LN patients compared to renal biopsies from healthy tissue or from patients with other types of kidney conditions [16]. Here, we observed a significantly altered N-glycome in the urine of LN patients compared to HC subjects. This included seven of the nine classes and 75% of the individual N-glycans (72 of the 96) detected in urine. Overall, the urine glycan profiles were more informative in regard to disease status as compared with the N-glycans determined in the patient matched serum samples. In the previous study of kidney biopsies, the largest difference was observed in the abundance of mannose-enriched N-glycans, which was higher in the kidneys of LN patients [16]. Conversely, we demonstrated a significant decrease in high mannose-containing N-glycans in the urine of LN patients in this study. The increase in mannose-containing N-glycans reported in the kidney [16] and the decrease we observed in high mannose-containing N-glycans in the urine may be due to differences in how the mannose-containing N-glycans were defined or grouped in the two analyses. Alternatively, differences in levels may be due to tissue versus secreted (into the urine). Future studies with matched urine and renal biopsies to compare levels within the same individuals using the same method of defining N-glycan classes are needed to address this question.

Age, sex, and body mass index (BMI) were associated with changes in the N-glycome [34,35,36,37,38,39]. Sex differences in the N-glycome reported in the literature are largely associated with IgG glycosylation. Pregnant women were reported to have higher levels of galactosylated and sialylated (anti-inflammatory) forms of IgG, which correlated with estrogen levels [34]. In a lupus study, estrogen was shown to alter IgG sialylation and induce an enzyme that adds sialic acid to N-glycans [40]. In our study, we observed that individual N-glycans in the urine at peaks 2361, 2289, and 2339 differed by sex, with higher levels of all three observed in males compared to females regardless of disease status. However, none of these peaks are associated with IgG. Although we also demonstrated differences in the serum N-glycome between LN and HC, no sex differences were observed. Identifying the proteins from which the three urine N-glycans were derived that differed between sexes is a future goal. Identifying the proteins from which these three N-glycans were derived may lead to a better understanding of sex bias mechanisms in LN. Importantly, inclusion of four of the urine N-glycans associated with LN in a model including total urine LacCers and biologic sex improved the AUC to 1.0, providing perfect separation of LN from HC. Thus, measuring GSLs and N-glycans in the urine could serve as biomarkers of disease. Similarly, inclusion of the serum N-glycans improved the AUC in this model in distinguishing LN patients from HC subjects. Future studies using longitudinal serum samples to survey N-glycans in lupus patients who have not or have developed nephritis to determine if specific serum N-glycans can predict which lupus patients are likely to develop nephritis are of interest.

As mentioned above, many of the differences in glycosylation previously reported relate to IgG. Fc N-linked glycosylation influences the pathogenicity of IgG. Loss of sialic acid and galactose residues from the IgG N-glycome is associated with pro-inflammatory effector functions and autoimmune diseases [9]. Changes in glycosylation of serum IgG autoantibodies in lupus including decreased sialylation and galactosylation were reported previously and an altered IgG glycome was associated with disease status [14]. Here, we observed significant differences in five N-glycans associated with IgG in the urine, and only two of those in the serum, of LN patients. In the urine, there was a significant increase in the degalactosylated (desialylated) glycans associated with pro-inflammatory IgG functions. This was coupled with a corresponding significant decrease in the glycan associated with anti-inflammatory IgG functions, resulting in extensive skewing towards more pro-inflammatory IgG effector functions. Interestingly, we did not observe a skewing to this extent in the serum. Since the serum and urine samples were collected from the same patient at the same visit, it is possible that in LN the more pro-inflammatory forms of IgG are deposited in the kidney (or other target organs), reducing their levels in the circulation. We hypothesize that LN patients with a more skewed pro-inflammatory IgG repertoire may have worse disease or may be more likely to develop nephritis and measuring these glycans could help inform treatment decisions. Longitudinal studies in SLE patients without nephritis that eventually develop nephritis may address whether IgG N-glycans could be used to monitor SLE patients to identify those who will eventually develop kidney disease. Moreover, identification of the individual N-glycans present in the urine may lead to a better understanding of disease mechanisms in LN since most of the proteins present in the urine are likely derived from the kidney.

There were some limitations to this study. The in vitro hRMC studies were performed using cells derived from one female donor and one male donor. Thus, it is possible that the differences observed are due to individual differences that are unrelated to the biologic sex of the donors. Additional analyses will need to be performed in a larger number of female and male donor-derived hRMCs to determine if there is a correlation between biologic sex and GSL metabolism (or N-glycome) and with pathological response. In addition, the female-derived hRMCs showed a significantly higher sensitivity in intracellular Ca^2+^ flux in response to LN sera, specifically at the low concentrations, which was similar to the higher release of CXCL5 and CCL5 in response to sera by the female-derived cells. Thus, future studies are needed to assess differences more globally (i.e., proliferation, apoptosis, or release of other cytokines, growth factors, or extracellular matrix proteins) in the effect of LN vs. HC serum. Another limitation is the small cohort size. GSLs and the N-glycome can vary based on age or can be influenced by ethnicity or environmental factors. The HC and LN groups in this study were matched closely in average age, but the groups were too small to adjust for age, ethnicity, race, or external/environmental factors. In particular, the LN group is 80% Black while the HC group is only 50% Black which may have impacted results since Blacks tend to have worse disease. Thus, the generalizability of our observations is limited. Future studies with a larger cohort will be needed to confirm our observations.

## 4. Materials and Methods

### 4.1. Human Samples and Ethics Statement

All results, except those in Supplemental Figure S2, analyzed stored urine and serum samples collected from the same subject at the same visit and included 20 healthy subjects (10 female and 10 male) and 20 lupus nephritis patients (10 female and 10 male). Lupus nephritis (LN) patients met the American College of Rheumatology classification for systemic lupus erythematosus (SLE) with nephritis and samples were collected during active disease. All except two LN patients had biopsy-confirmed nephritis. Healthy subjects (healthy controls, HC) did not have documented autoimmunity, renal disease, an active infection, or an ongoing pregnancy at the time of sample collection. Patient demographics and relevant clinic measures are provided in Table 1. Urine Protein:Creatinine ratio (UPCr), eGFR, C3, and C4 measures were missing for twelve of the HC subjects. The reported values in Table 1 are representative of the eight for whom measures were available. For the autoantibodies, LN patients are reported in Table 1 as having ever been positive for anti-Sm and anti-RNP and positive for anti-dsDNA at the time of sample collection for the samples used in this study. Anti-Sm and anti-RNP measures were not available for two LN patients. For analyses in Supplemental Figure S2, stored serum samples from 12 HC, 12 LN patients at the time of inactive disease, and 12 LN patients at the time of active disease were used to stimulate mesangial cells as described below. Inclusion and exclusion criteria for HC and LN subjects were the same as described above.

### 4.2. Cell Culture

Two lots of primary human renal mesangial cells (hRMCs) were commercially obtained from ScienCell (Carlsbad, CA, USA). Each lot was derived from one individual, a 21-week gestation female (referred to as “female-derived”) and a 22-week gestation male (referred to as “male-derived”) who presumably did not have disease. Cells were negative for HIV-1, HBV, HCV, mycoplasma, bacteria, yeast, and fungi. hRMCs were maintained on poly-l-lysine coated flasks in complete growth mesangial cell media (MCM) (1% penicillin/streptomycin and 1% mesangial cell growth supplement) that was supplemented with 2% FBS in a humidified 5% CO_2_ atmosphere at 37 **°**C according to manufacturer’s recommendations (ScienCell). Cells at passages 5 or 6 were used for experiments.

### 4.3. Lipid Analyses

Glycosphingolipids hexosylceramides (HexCers) and lactosylceramides (LacCers) of individual chain lengths C16, C18, C18:1, C20, C20:1, C22, C22:1, C24, C24:1, C26, and C26:1 were quantified by the Lipidomics Core Facility at MUSC as we described previously [7,20,41]. The most highly expressed (“major”) chain lengths in urine, serum, and hRMCs were C16, C22, C24, and C24:1 and quantified levels of these four chain lengths are provided on the graphs. The reported “total” HexCers or LacCers are the sum measures of all 11 chain lengths listed above. For urine, equivalent volumes of urine from each subject were provided and lipid measures were normalized to urine creatinine. Creatinine levels in all urine samples were measured in our laboratory in the same assay by the Jaffe picric acid method [42]. For serum, equivalent volumes of serum from each subject were provided to the core facility and lipids are presented as pmol of lipid per ml of serum. For the hRMCs, lipids were measured in cell pellets and are presented as pmol of lipids normalized to relative cell viability as measured by alamar blue just prior to collecting the cells.

### 4.4. N-Glycan Analyses

The N-glycan analysis of urine and serum was performed as previously described [43,44]. Serum was diluted 1:2 in 100 mM sodium bicarbonate pH 8.0 and 1 µL spotted on a Nexterion Slide H amine-reactive hydrogel-coated glass slide from Applied Microarrays (Tempe, AZ, USA). Urine samples were buffer exchanged in phosphate buffered saline and concentrated using a 0.5 mL Amicon 10,000 MW centrifugation tube prior to spotting [43]. After a 1-h incubation, salts and lipids were removed using a Carnoy’s solution (10% glacial acetic acid, 30% chloroform, 60% ethanol) wash. The samples were then sprayed with the enzyme peptide N-glycosidase F (PNGase F PRIME, N-Zymes Scientific, Doylestown, PA, USA) and incubated for 2 h to cleave N-glycans from the captured glycoproteins. Finally, an α-cyano-4-hydroxycinnamic acid (CHCA) matrix was sprayed onto the slides before performing MALDI-IMS using a Bruker 7T SolariX MALDI-FTICR mass spectrometer for serum and a Bruker MALDI-QTOF timsTOF fleX mass spectrometer for urine.

For cells, N-glycans were quantified as we previously described [18,45]. Briefly, female- or male-derived hRMCs cells were seeded at 6000 cells per well in duplicate wells on 8-well LabTekII chamber slides (Electron Microscopy Sciences, Hatfield, PA, USA). Wells with no cells (media only) were used to determine background levels from media. Cells were washed with PBS, fixed for 30 min in 10% buffered formalin, washed, and stored in PBS until analysis. N-glycans released by PNGase F digestion were detected by MALDI-FTICR as previously reported [18,45].

N-glycan peaks were analyzed using SCiLS Lab (v. 2021b) software (Bruker, Billerica, MA, USA) as previously reported [43]. Mass spectra were normalized to total ion current. Peaks were selected for N-glycans based on theoretical and established mass values, and maximum mean values for each peak were included in subsequent analyses. Background signal in the blank well was subtracted from each N-glycan measurement to obtain an absolute intensity. Relative intensities of N-glycans were calculated (absolute intensity divided by the intensity of all N-glycans detected in each sample). This accounted for protein concentration differences that could lead to higher signal intensities from sample to sample and allow for detection of low-abundance N-glycans. The sum of relative intensities of the individual N-glycans in a specific class was used to calculate each N-glycan classes (Bi-, Tri-, or Tetra-antennary, bisecting, and hybrid). In addition, each N-glycan was placed into a group based on the absence or presence of mannose, sulfate, sialic acid, or fucose. The sum of relative intensities in these classes/groups were then compared. Sialylated or sulfated N-glycans with multiple sodiated species were included together when comparing the intensities of individual N-glycans across samples. A cumulative peak and structure list of N-glycans used in the statistical analyses is provided in Supplemental Table S1.

### 4.5. Cytokine Release Experiments

Female-derived or male-derived hRMCs described above were serum-starved for three hours in serum-free complete MCM (without FBS supplementation) when ~80% confluent. Human sera were then added to a final concentration of 5%, incubated for three hours, and refed with fresh serum-free complete MCM. Serum from a single individual was used for all experiments. All treatments were performed in duplicate or triplicate. Media was collected from hRMCs following incubation with human sera. Cell viability was then measured using the Alamar Blue assay (Invitrogen/ThermoFisher, Waltham, MA, USA) following the manufacturer’s instructions. ELISA kits from Biolegend (San Diego, CA, USA) were used to measure CCL5 (RANTES) or CXCL5 according to the manufacturer protocol. Relative cell viability (per well) with respect to untreated cells was used to normalize measured cytokine levels per well. For the individual serum analyses, replicates for each serum donor were averaged. The averages for each serum donor are shown on the graphs as individual points.

### 4.6. Intracellular Ca^2+^ Analyses

The male- and female-derived hRMCs described above were used in confocal fluorescent experiments. The experiments were performed similarly to the previously described protocol [46]. Briefly, cells were grown on glass-bottom dishes (#0 glass, Mattek, Ashland, MA, USA) and loaded with fluorescent Ca^2+^ indicator Fluo-8 AM (AAT Bioquest, Pleasanton, CA, USA). After loading, cells were rinsed and media was replaced with an extracellular solution containing in mM: 2 CaCl_2_, 145 NaCl, 2 MgCl_2_, 4.5 KCl, 10 HEPES, pH 7.4 adjusted by NaOH. Confocal imaging was performed using the Leica TCS SP5 laser scanning microscope equipped with an HCX Plan Apochromat 40× 1.25 NA oil objective (Leica Microsystems Inc., Deerfield, IL, USA). Maximum amplitude of intracellular Ca^2+^ transient in individual hRMCs was obtained in response to the application of human sera from LN or HC patients in concentrations from 0.01 to 5% (at least three separate experiments per group). The data were analyzed using ImageJ (NIH) and summarized in OriginPro 2021b software (OriginLab, Northampton, MA, USA).

### 4.7. Statistical Analyses

Descriptive statistics were determined for participant characteristics by LN status. Differences in patient characteristics for categorical variables were examined using Fisher’s exact tests and for continuous or ordinal variables were examined using 2-sample t-tests or Wilcoxon rank sum tests as appropriate.

Comparisons of LacCers or N-glycans in urine and serum by disease status and by biologic sex were examined for associations using a series of linear mixed models. Fixed effects in the models included disease status and biologic sex. We also considered the disease status by biologic sex interaction but only retained if it was statistically significant. For the N-glycans, all models also included a random batch effect to control for correlation between samples run in the same batch. *p*-values for the associations between LacCers or N-glycans with disease status or with sex were adjusted using FDR to control for multiple testing. All FDR q-values < 0.05 are considered meaningful. We also evaluated if including individual N-glycans improved prediction of disease status in a model including total urine LacCers and biologic sex using the likelihood ratio test to determine if inclusion of glycans improved prediction and area under the receiver operating characteristics curve (AUC) to examine improvement in ability to discriminate between LN patients and HC subjects.

Differences in CXCL5 and CCL5 production between hRMCs treated with sera from healthy controls versus lupus nephritis were also examined. Additional factors considered included serum source (male or female donors) and cell line biologic sex. Differences between groups in CXCL5 or CCL5 expression were evaluated using a linear model approach. Models included main effects for disease status of the serum donor (HC vs. LN), sex of the serum donor, and sex of the hRMC donor. Two-way interactions between cell type, derived sex, and serum sex were considered but were not significant and thus only main effects were considered. Differences by disease status, serum sex, and derived cell sex were estimated using linear contrasts. *p*-values were Bonferroni adjusted for the three pairwise comparisons. Model assumptions were checked graphically and transformations were considered as needed.

## 5. Conclusions

This study demonstrates that altered GSLs and N-glycosylation could serve as effective biomarkers of LN, particularly in urine, and that elevated cellular GSLs levels in the female-derived hRMCs were associated with a greater response (higher levels of cytokine secretion and intracellular Ca^2+^ flux) to human sera. GSLs and N-glycans also warrant further investigation as potential predictive biomarkers of LN and future studies are needed to determine if the elevated levels of GSLs in the female-derived cells are due to sex differences or other individual donor differences. Elucidating the mechanisms by which GSLs and an altered N-glycome contribute to disease, and specifically the response of renal cells to external stimuli, could provide a better understanding of disease pathology.

## Figures and Tables

**Table 1:** Demographics and clinical measures of healthy controls (HC) and lupus nephritis (LN) patients.

	HC (N = 20)	LN (N = 20)	*p*
Sex, male, n (%)	10 (50)	10 (50)	1.000
Age, mean (SD)	34.0 (10.3)	33.2 (11.2)	0.824
Race, n (%)			0.041
Black	10 (50)	16 (80)	
White	10 (50)	3 (15)	
Other	0 (0)	1 (5)	
Estimated Glomerular Filtration Rate, mean (SD)	103.9 (23.9)	93.0 (51.9)	0.455
Urine Creatinine, mg/mL (SD)	1.46 (1.14)	1.24 (0.82)	0.484
Urine Protein: Creatinine, median (IQR)	0.055 (0.033)	1.69 (3.48)	<0.001
Nephritis Class, n (%)			N/A
I	N/A	2 (10)	
II	N/A	2 (10)	
III, IV	N/A	8 (40)	
III + V, IV + V	N/A	3 (15)	
V	N/A	3 (15)	
No biopsy/missing	N/A	2 (10)	
SLEDAI, mean (SD)	N/A	11.85 (5.6)	N/A
Anti-dsDNA, n positive (%)	N/A	15 (75)	N/A
Anti-Sm, n positive (%)	N/A	10 (56)	N/A
Anti-RNP, n positive (%)	N/A	11 (61)	N/A
C3 Complement, mean (SD)	151.0 (16.1)	85.8 (21.5)	<0.001
C4 Complement, mean (SD)	33.9 (11.5)	23.0 (8.18)	0.034

**Table 2:** AUCs, likelihood ratio test (LRT) *p*-values, and differences in AUC of adding urine N-glycans to a “null” model. The null model included only total urine LacCers and biologic sex. Urine LacCers was natural log transformed prior to fitting the models to meet statistical assumptions. * Inclusion of this glycan yielded perfect separation of the cases and controls.

	AIC	LRT *p*-Value	AUC (95% CI)	Δ AUC (95% CI)
Null Model	43.12	0.0020	0.870 (0.748, 0.992)	
2669 *	8	0.9970	1 (1, 1)	0.13 (0.008, 0.252)
1419 *	8	0.9976	1 (1, 1)	0.13 (0.008, 0.252)
1581 *	8	0.9987	1 (1, 1)	0.13 (0.008, 0.252)
Mannose *	8	0.9987	1 (1, 1)	0.13 (0.008, 0.252)
1485	33.92	0.0081	0.932 (0.859, 1.00)	0.062 (−0.031, 0.156)
1853	30.1	0.0081	0.945 (0.866, 1.00)	0.075 (−0.025, 0.175)
2174	32.47	0.0085	0.943 (0.876, 1.00)	0.073 (−0.036, 0.181)
1866	31.85	0.0095	0.948 (0.88, 1.00)	0.078 (−0.023, 0.178)
2122	33.54	0.0107	0.943 (0.872, 1.00)	0.073 (−0.047, 0.192)
2158	28.28	0.0108	0.958 (0.903, 1.00)	0.088 (−0.023, 0.198)
1996	31.62	0.0111	0.948 (0.887, 1.00)	0.078 (−0.026, 0.181)
2377	25.13	0.0119	0.970(0.923, 1.00)	0.100 (−0.019, 0.219)
2361	34	0.0124	0.938 (0.860, 1.00)	0.068 (−0.018, 0.153)
Tetraantennary	33.13	0.0130	0.938 (0.860, 1.00)	0.068 (−0.054, 0.189)
2012	34.74	0.0131	0.917 (0.830, 1.00)	0.047 (−0.062, 0.157)
2967	31.54	0.0159	0.943 (0.861, 1.00)	0.073 (−0.042, 0.187)
2289	36.13	0.0170	0.915 (0.828, 1.00)	0.045 (−0.059, 0.149)
Sialylation	35.54	0.0182	0.948 (0.874, 1.00)	0.078 (−0.018, 0.173)
1831	34.34	0.0212	0.932 (0.846, 1.00)	0.062 (−0.014, 0.139)
2056	37.2	0.0225	0.915 (0.829, 1.00)	0.045 (−0.025, 0.115)
1704	33.24	0.0242	0.955 (0.886, 1.00)	0.085 (−0.011, 0.181)
1809	37.7	0.0250	0.917 (0.833, 1.00)	0.047 (−0.036, 0.131)
3770	37.38	0.0264	0.902 (0.805, 1.00)	0.032 (−0.062, 0.127)
2632	38.17	0.0289	0.907 (0.813, 1.00)	0.037 (−0.035, 0.110)
2487	38.8	0.0311	0.897 (0.806, 0.989)	0.027 (−0.065, 0.120)
2267	37.86	0.0314	0.915 (0.830, 1.00)	0.045 (−0.038, 0.128)
1257	20.36	0.0346	0.985 (0.959, 1.00)	0.115 (−0.005, 0.235)
2245	38.88	0.0363	0.902 (0.807, 0.998)	0.032 (−0.034, 0.099)
2852	38.39	0.0367	0.912 (0.823, 1.00)	0.042 (−0.068, 0.153)
1079	39.5	0.0372	0.900 (0.793, 1.00)	0.030 (−0.067, 0.127)
2221	39.48	0.0389	0.902 (0.807, 0.998)	0.032 (−0.047, 0.112)
2638	38.44	0.0406	0.910 (0.809, 1.00)	0.040 (−0.040, 0.120)
3333	38.94	0.0412	0.897 (0.800, 0.995)	0.027 (−0.070, 0.125)
2287	38.33	0.0445	0.902 (0.808, 0.997)	0.032 (−0.040, 0.105)
1663	39.57	0.0477	0.885 (0.774, 0.996)	0.015 (−0.032, 0.062)
1891	19.61	0.0557	0.988 (0.965, 1.00)	0.118 (−0.001, 0.236)
1743	19.12	0.0567	0.985 (0.957, 1.00)	0.115 (0.005, 0.225)
2945	40.2	0.0586	0.897 (0.801, 0.994)	0.027 (−0.032, 0.087)
1905	21.5	0.0604	0.983 (0.954, 1.00)	0.113 (0.001, 0.224)
1444	37.27	0.0657	0.920 (0.831, 1.00)	0.05 (−0.038, 0.138)
2465	41.09	0.0714	0.890 (0.792, 0.988)	0.02 (−0.053, 0.093)
1960	41.41	0.0741	0.887 (0.774, 1.00)	0.017 (−0.038, 0.073)
2523	37.65	0.0813	0.907 (0.809, 1.00)	0.037 (−0.024, 0.099)
3144	40.32	0.0826	0.897 (0.800, 0.995)	0.027 (−0.05, 0.105)
1647	41.44	0.0864	0.895 (0.798, 0.992)	0.025 (−0.04, 0.09)
3646	41.69	0.0948	0.887 (0.787, 0.988)	0.017 (−0.043, 0.078)
2654	41.54	0.1038	0.887 (0.779, 0.996)	0.017 (−0.059, 0.094)
1954	42.03	0.1056	0.892 (0.790, 0.995)	0.022 (−0.036, 0.081)
2610	41.41	0.1125	0.885 (0.777, 0.993)	0.015 (−0.038, 0.068)
2028	42.3	0.1158	0.893 (0.778, 1.00)	0.023 (−0.024, 0.069)
sulfation	42.4	0.1233	0.900 (0.793, 1.00)	0.03 (−0.016, 0.076)
2163	42.2	0.1234	0.882 (0.774, 0.991)	0.012 (−0.062, 0.087)
2923	42.01	0.1353	0.880 (0.774, 0.986)	0.01 (−0.052, 0.072)
1814	42.61	0.1363	0.885 (0.786, 0.984)	0.015 (−0.061, 0.091)
2304	42.78	0.1480	0.887 (0.784, 0.991)	0.017 (−0.057, 0.092)
3092	42.8	0.1579	0.873 (0.754, 0.991)	0.003 (−0.034, 0.039)
1850	42.93	0.1609	0.88 (0.771, 0.989)	0.01 (−0.059, 0.079)
3193	42.38	0.1649	0.885 (0.778, 0.992)	0.015 (−0.042, 0.072)
3113	42.56	0.1651	0.882 (0.769, 0.996)	0.012 (−0.017, 0.042)
3004	42.8	0.1815	0.878 (0.760, 0.995)	0.008 (−0.03, 0.045)
2435	43.22	0.1838	0.875 (0.769, 0.981)	0.005 (−0.06, 0.07)
3093	43.2	0.1933	0.865 (0.740, 0.990)	−0.005 (−0.039, 0.029)
2393	43.16	0.2030	0.880 (0.769, 0.991)	0.010 (−0.042, 0.062)
fucosylation	43.35	0.2073	0.885 (0.780, 0.990)	0.015 (−0.044, 0.074)
2100	43.33	0.2168	0.870 (0.754, 0.986)	0.00 (−0.048, 0.048)
3384	43.18	0.2193	0.873 (0.755, 0.99)	0.003 (−0.044, 0.049)
triantennary	43.7	0.2642	0.870 (0.747, 0.993)	0.00 (−0.052, 0.052)
bisect	43.82	0.2776	0.882 (0.774, 0.991)	0.012 (−0.045, 0.07)
2319	43.58	0.2828	0.882 (0.772, 0.993)	0.012 (−0.036, 0.061)
1875	44.09	0.3257	0.882 (0.769, 0.996)	0.012 (−0.017, 0.042)
2383	44.55	0.4570	0.882 (0.767, 0.998)	0.012 (−0.019, 0.044)
2413	44.69	0.5224	0.863 (0.739, 0.986)	−0.007 (−0.041, 0.026)
1773	44.75	0.5600	0.875 (0.756, 0.994)	0.005 (−0.016, 0.026)
1611	44.83	0.5933	0.877 (0.762, 0.993)	0.007 (−0.02, 0.035)
2594	44.87	0.6229	0.877 (0.759, 0.996)	0.007 (−0.019, 0.034)
2339	44.89	0.6393	0.875 (0.752, 0.998)	0.005 (−0.019, 0.029)
2341	44.97	0.7073	0.875 (0.758, 0.992)	0.005 (−0.019, 0.029)
2391	45.1	0.9027	0.873 (0.750, 0.995)	0.003 (−0.004, 0.009)

**Table 3:** AUCs, likelihood ratio test (LRT) *p*-values, and differences in AUC of adding serum N-glycans to a “null” model (same null model as in Table 2).

	AIC	LRT *p*-Value	AUC (95% CI)	D AUC
Null Model	43.12	0.002	0.870 (0.748, 0.992)	
mannose	29.08	0.012	0.955 (0.900, 1.00)	0.085 (−0.016, 0.186)
1743	29.71	0.013	0.953 (0.897, 1.00)	0.083 (−0.013, 0.178)
1581	30.74	0.008	0.950 (0.887, 1.00)	0.080 (−0.024, 0.184)
sulfation	32.53	0.006	0.945 (0.875, 1.00)	0.075 (−0.031, 0.181)
1419	32.62	0.006	0.943 (0.876, 1.00)	0.073 (−0.030, 0.175)
triantennary	32.94	0.013	0.935 (0.867, 1.00)	0.065 (−0.041, 0.171)
1809	33.3	0.009	0.938 (0.868, 1.00)	0.068 (−0.039, 0.174)
2275	33.33	0.016	0.927 (0.852, 1.00)	0.057 (−0.041, 0.156)
2540.1	33.69	0.007	0.935 (0.864, 1.00)	0.065 (−0.023, 0.153)
2028	34.96	0.02	0.917 (0.834, 1.00)	0.047 (−0.052, 0.147)
1444	35.96	0.028	0.917 (0.834, 1.00)	0.047 (−0.044, 0.139)
1905	36.12	0.015	0.935 (0.855, 1.00)	0.065 (−0.009, 0.139)
1136	36.24	0.019	0.910 (0.823, 0.997)	0.040 (−0.054, 0.134)
tetraantennary	36.79	0.015	0.915 (0.830, 1.00)	0.045 (−0.049, 0.139)
1257	36.87	0.014	0.920 (0.831, 1.00)	0.050 (−0.044, 0.144)
2319	37.69	0.026	0.900 (0.809, 0.991)	0.030 (−0.072, 0.132)
2122	38.32	0.024	0.910 (0.826, 0.994)	0.040 (−0.053, 0.133)
2633.1	39.17	0.043	0.900 (0.809, 0.991)	0.030 (−0.060, 0.120)
2523.1	39.2	0.036	0.895 (0.799, 0.991)	0.025 (−0.059, 0.109)
2231	40.01	0.053	0.897 (0.803, 0.992)	0.027 (−0.050, 0.105)
2393	40.16	0.04	0.905 (0.813, 0.997)	0.035 (−0.045, 0.115)
2341	40.65	0.066	0.895 (0.799, 0.991)	0.025 (−0.050, 0.100)
biantennary	40.83	0.053	0.897 (0.788, 1.00)	0.027 (−0.036, 0.091)
1647	41.87	0.091	0.890 (0.780, 1.00)	0.020 (−0.037, 0.077)
2968.1	42.67	0.15	0.880 (0.774, 0.986)	0.010 (−0.059, 0.079)
2655.1	42.75	0.158	0.875 (0.767, 0.983)	0.005 (−0.071, 0.081)

## Data Availability

All data supporting the conclusions of this study are available upon request to the corresponding author.

## Supplementary Materials

The supporting information can be downloaded at https://www.mdpi.com/article/10.3390/ijms242216490/s1.
