# Supplementary material for: Metabolic Markers and Association of Biological Sex in Lupus Nephritis
Source: Int J Mol Sci. 2023 Nov 18;24(22):16490. doi: 10.3390/ijms242216490 (PMC10671813; doi:10.3390/ijms242216490)
Supplement: Supplementary file 1 [file ijms-24-16490-s001.zip › ijms-2676084-supplementary.pdf]

Supplemental Methods, Figures, Tables

Methods for supplemental Figure S2

For this experiment, media from female-derived hRMCs were seeded in a 96-well plate, serum-starved, and incubated with donor serum from each of 12 individuals from the following three groups of donors: HC, LN with inactive disease, LN with active disease. These sera were from a separate cohort from the sera described in the main text. Incubations were performed in triplicate for each donor. Media was collected from each well and used for the individual IL-6 and MCP-1 ELISAs and screening a cytokine array as follows. For IL-6 and MCP-1 ELISAs (BioLegend), media from each well was run on the ELISAs, triplicates for each individual were averaged and graphed as an independent point (Figure S1A). The remaining media was then pooled to collect sufficient volume to screen a cytokine array. Two pools for each group (replicate wells of 6 individuals per group) was obtained and used to screen a 71-cytokine array (Eve Technologies, Calgary, Canada). The two samples per group were averaged and the means +SD are presented. Cytokine levels were normalized to cell viability using the alamar blue assay.

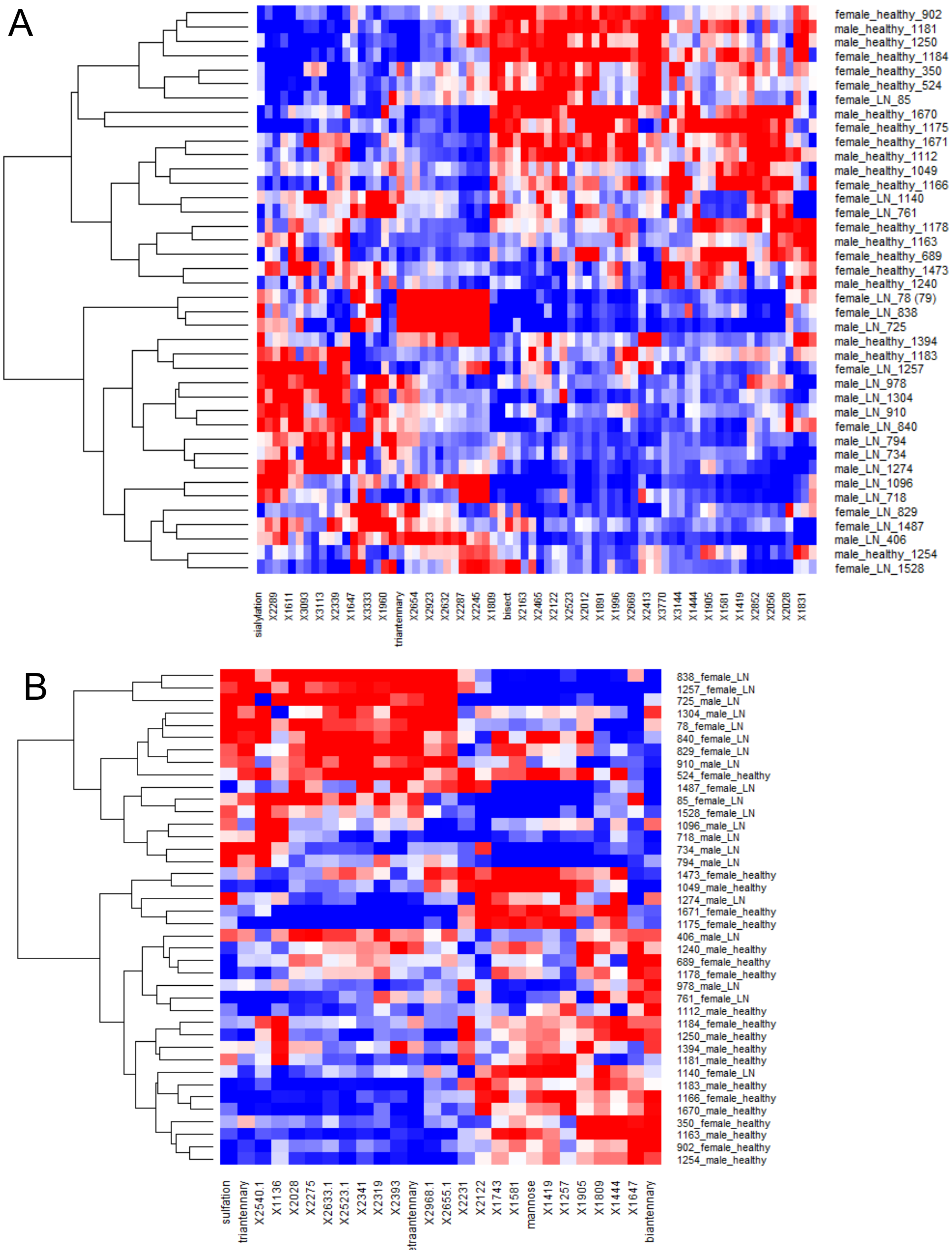

Figure S1. Heatmaps of N-glycans associated with disease status. Heatmaps of the 72 urine N-glycans (A) and 26 serum N-glycans (B) associated with disease status.

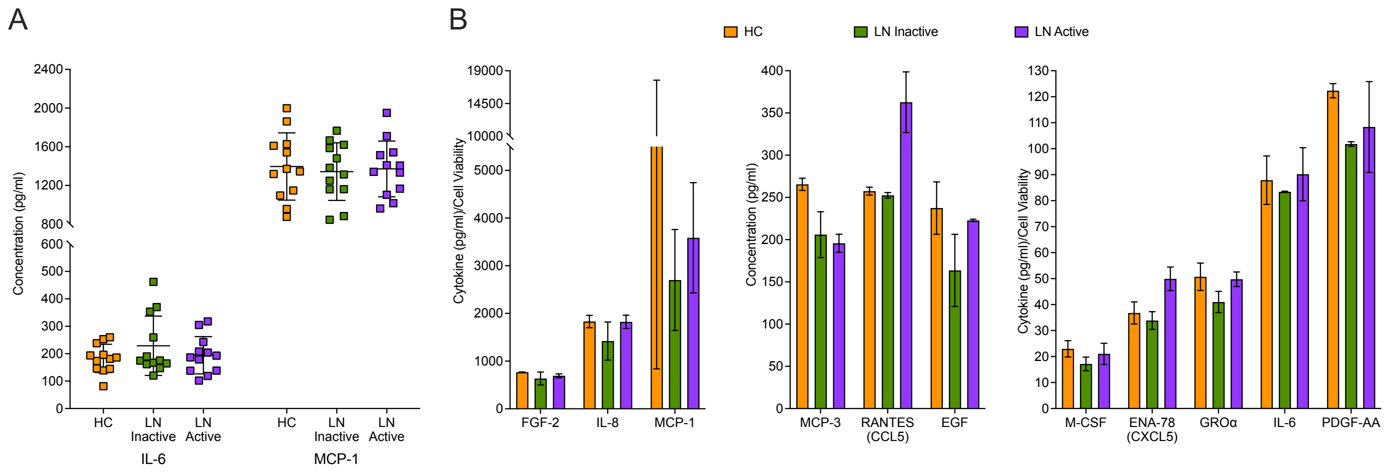

Figure S2. Cytokines released from female-derived primary human renal mesangial cells (hRMCs) in response to human sera. A) Female-derived hRMCs were stimulated with 10% human sera from 12 healthy control subjects (HC), 12 lupus nephritis patients with inactive disease (LN Inactive), or 12 lupus nephritis patients with active disease (LN Active). Treatments were performed in triplicate. The average of the triplicates for each subject is plotted on the graph. IL-6 and MCP-1 released into the media were quantified by ELISA and normalized to cell viability. B) Media was pooled from the cells treated in (A) within each group as described in the supplemental methods and used to screen an array containing 71 cytokines. Graphed cytokines include the most highly expressed cytokines. Levels were normalized to cell viability and levels measured in the media from untreated cells were subtracted. Since these analyses were from a single experiment, statistical analyses were not performed; however, CCL5 and CXCL5 showed trends of higher levels in the media of cells treated with LN Active sera compared to LN Inactive sera or HC sera. Thus, these two cytokines were chosen for analyses in subsequent experiments.

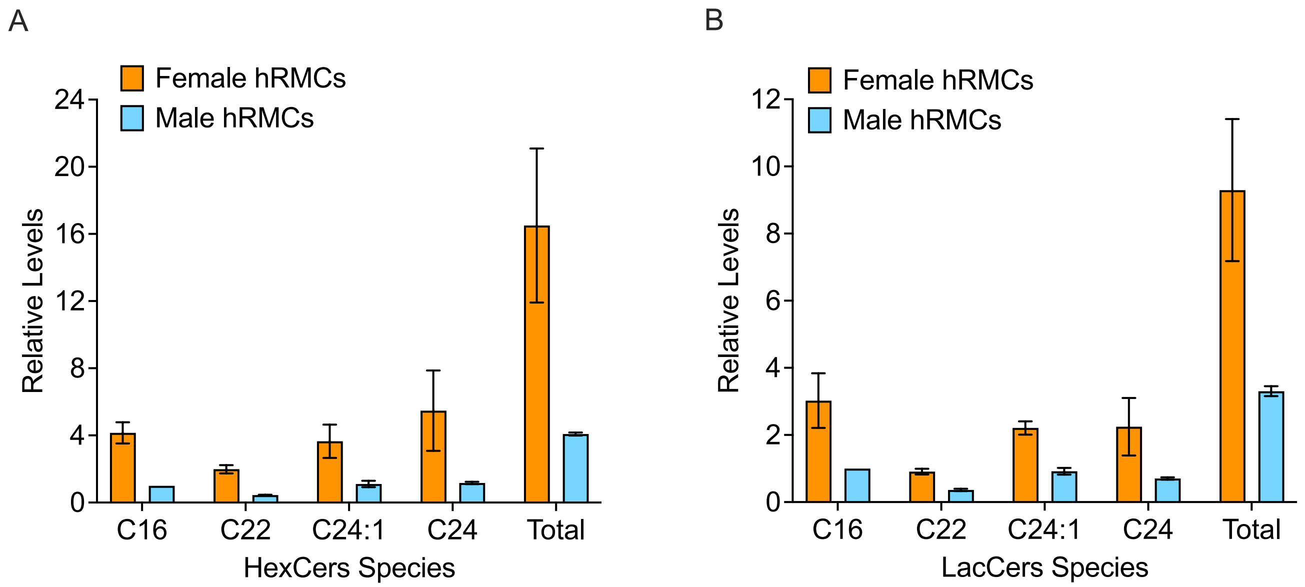

Figure S3. Female-derived hRMCs had higher levels of HexCers and LacCers. Female-derived and male-derived hRMCs were maintained in culture as indicated in the Methods section and one 10 mm plate of cells collected at passages 5 and 6 after measuring cell viability using alamar blue assay. HexCers (A) and LacCers (B) were measured for each passage and normalized to cell viability. Relative levels were calculated by setting the levels for HexCer or LacCer C16 to 1.0 for the male-derived cells and all other levels for both the male- and female-derived cells are relative to it within each passage. Normalized levels for each passage were averaged. Means +SD are presented. Statistical analyses were not performed since the measures were performed in hRMCs from only one female donor and one male donor.

Supplementary Table 1. Cumulative peak list of detected N-glycans in serum, urine, and cells.

Theoretical Mass	Mass in Tables or Figures	Composition	Putative Structure	+SO4	+Na	Glycosylation Class
933.371	933	Hex3HexNAc2 + 1Na	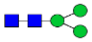	0	1	
1079.375	1079	Hex3dHex1HexNAc2 + 1Na	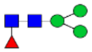	0	1	Fuc
1095.370	1095	Hex4HexNAc2 + 1Na	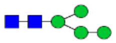	0	1	Man
1136.396	1136	Hex3HexNAc3 + 1Na	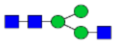	0	1	
1257.423	1257	Hex5HexNAc2 + 1Na	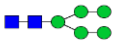	0	1	Man
1282.454	1282	Hex3dHex1HexNAc3 + 1Na	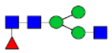	0	1	Fuc
1298.449	1298	Hex4HexNAc3 + 1Na	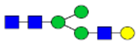	0	1	
1339.476	1339	Hex3HexNAc4 + 1Na	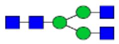	0	1	Bia
1419.476	1419	Hex6HexNAc2 + 1Na	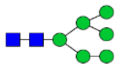	0	1	Man
1444.507	1444	Hex4dHex1HexNAc3 + 1Na	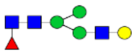	0	1	Fuc
1460.502	1460	Hex5HexNAc3 + 1Na	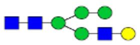	0	1	Hyb

1485.534	1485	Hex3dHex1HexNAc4 + 1Na	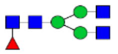	0	1	Fuc, Bia
1501.529	1501	Hex4HexNAc4 + 1Na	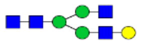	0	1	Bia
1581.528	1581	Hex7HexNAc2 + 1Na	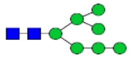	0	1	Man
1606.560	1606	Hex5dHex1HexNAc3 + 1Na	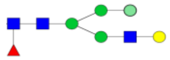	0	1	Fuc, Hyb
1611.527	1611	Hex4HexNAc3NeuAc1 + 2Na	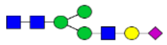	0	2	Sia
1622.555	1622	Hex6HexNAc3 + 1Na	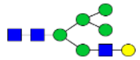	0	1	Hyb
1647.587	1647	Hex4dHex1HexNAc4 + 1Na	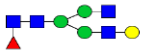	0	1	Fuc, Bia
1663.581	1663	Hex5HexNAc4 + 1Na	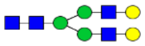	0	1	Bia
1688.613	1688	Hex3dHex1HexNAc5 + 1Na	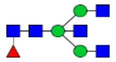	0	1	Fuc, Bis
1704.608	1704	Hex4HexNAc5 + 1Na	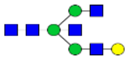	0	1	Bis
1743.581	1743	Hex8HexNAc2 + 1Na	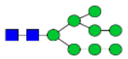	0	1	Man
1751.597	1751	Hex5HexNAc3NeuAc1 + 1Na	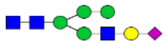	0	1	Hyb, Sia
1757.585	1757	Hex4dHex1HexNAc3NeuAc1 + 2Na	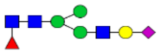	0	2	Fuc, Sia

1773.579	1773	Hex5HexNAc3NeuAc1 + 2Na	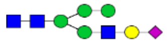	0	2	Hyb, Sia
1809.639	1809	Hex5dHex1HexNAc4 + 1Na	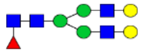	0	1	Fuc, Bia
1814.606	1814	Hex4HexNAc4NeuAc1 + 2 Na	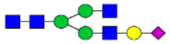	0	2	Bia, Sia
1815.559	1815	Hex4dHex1HexNAc3NeuAc1 + $1SO_4$ + 1Na	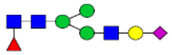	1	1	Fuc, Sia, Sul
1825.634	1825 / 1826	Hex6HexNAc4 + 1Na	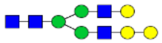	0	1	Bia, Alp
1831.554	1831	Hex5HexNAc3NeuAc1 + $1SO_4$ + 1Na	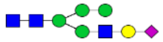	1	1	Hyb, Sia, Sul
1850.666	1850	Hex4dHex1HexNAc5 + 1Na	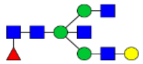	0	1	Fuc, Bis
1853.536	1853	Hex5HexNAc3NeuAc1 + $1SO_4$ + 2Na	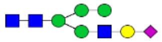	1	2	Hyb, Sia, Sul
1866.661	1866	Hex5HexNAc5 + 1Na	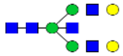	0	1	Bis
1875.518	1875	Hex5HexNAc3NeuAc1 + $1SO_4$ + 3Na	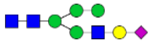	1	3	Hyb, Sia, Sul
1891.692	1891	Hex3dHex1HexNAc6 + 1Na	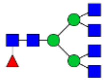	0	1	Fuc, Tet
1905.634	1905 / 1906	Hex9HexNAc2 + 1Na	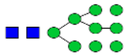	0	1	Man
1911.578	1910 / 1911	Hex5dHex1HexNAc4 + 1SO4 + 2Na	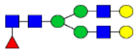	1	2	Fuc, Bia, Sul

1935.632	1954 / 1955	Hex6HexNAc3NeuAc1 + 2Na	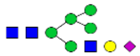	0	2	Hyb, Sia
1960.664	1960	Hex4dHex1HexNAc4NeuAc1 + 2Na	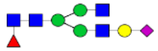	0	2	Fuc, Bia, Sia
1976.659	1976	Hex5HexNAc4NeuAc1 + 2Na	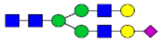	0	2	Bia, Sia
1996.724	1996	Hex4dHex2HexNAc5 + 1Na	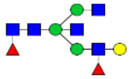	0	1	Fuc, Bis
2012.719	2012	Hex5dHex1HexNAc5 + 1Na	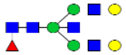	0	1	Fuc, Bis
2018.639	2018	Hex4dHex1HexNAc4NeuAc1 + 1SO_4 + 1Na	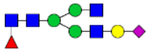	1	1	Fuc, Bia, Sia, Sul
2028.714	2028	Hex6HexNAc5 + 1Na	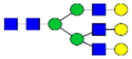	0	1	Tri
2037.750	2037	Hex3dHex2HexNAc6 + 1Na	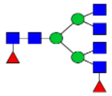	0	1	Fuc, Tet
2056.616	2056 / 2057	Hex5HexNAc4NeuAc1 + 1SO_4 + 2Na	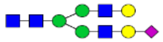	1	2	Bia, Sia, Sul
2062.624	2062	Hex4dHex1HexNAc4NeuAc1 + 1SO_4 + 3Na	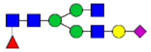	1	3	Fuc, Bia, Sia, Sul
2067.654	2067 / 2068	Hex10HexNAc2 + 1Na	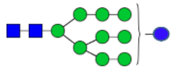	0	1	Man
2100.735	2100	Hex5dHex1HexNAc4NeuAc1 + 1Na	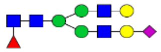	0	1	Fuc, Bia, Sia
2122.717	2122	Hex5dHex1HexNAc4NeuAc1 + 2Na	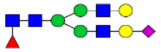	0	2	Fuc, Bia, Sia

2157.756	2157	Hex5HexNAc5NeuAc1 + 1Na	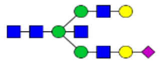	0	1	Bis, Sia
2158.777	2158	Hex5dHex2HexNAc5 + 1Na	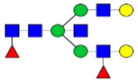	0	1	Fuc, Bis
2163.743	2163	Hex4dHex1HexNAc5NeuAc1 + 2Na	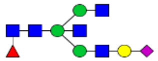	0	2	Fuc, Bis, Sia
2174.772	2174	Hex6dHex1HexNAc5 + 1Na	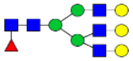	0	1	Fuc, Tri
2180.692	2180	Hex5dHex1HexNAc4NeuAc1 + 1Na	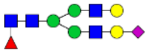	1	1	Fuc, Bia, Sia, Sul
2215.798	2215	Hex5dHex1HexNAc6 + 1Na	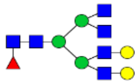	0	1	Fuc, Tet
2221.753	2221	Hex4dHex1HexNAc5NeuAc1 + 1SO_4 + 1Na	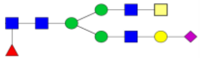	1	1	Fuc, Bia, Sia, Sul
2231.793	2231	Hex6HexNAc6 + 1Na	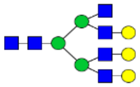	0	1	Tet
2245.772	2245	Hex5HexNAc4NeuAc2 + 1Na	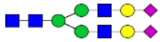	0	1	Bia, Sia
2259.738	2259	Hex8HexNAc3NeuAc1 + 2Na	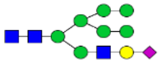	0	2	Hyb, Sia
2267.754	2267	Hex5HexNAc4NeuAc2 + 2Na	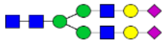	0	2	Bia, Sia
2276.710	2275 / 2276	Hex6dHex1HexNAc5 + 1SO_4 + 2Na	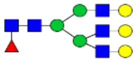	1	2	Fuc, Tri, Sul

2287.819	2287	Hex4dHex2HexNAc5NeuAc1 + 1Na	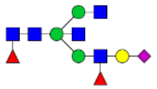	0	1	Fuc, Bis, Sia
2289.736	2289	Hex5HexNAc4NeuAc2 + 3Na	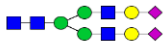	0	3	Bia, Sia
2303.814	2303	Hex5dHex1HexNAc5NeuAc1 + 1Na	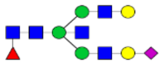	0	1	Fuc, Bis, Sia
2304.835	2304	Hex5dHex3HexNAc5 + 1Na	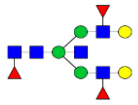	0	1	Fuc, Bis
2319.809	2319	Hex6HexNAc5NeuAc1 + 1Na	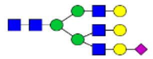	0	1	Tri, Sia
2325.796	2325	Hex5dHex1HexNAc5NeuAc1 + 2Na	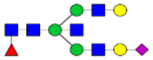	0	2	Fuc, Tri, Sia
2339.695	2339	Hex8HexNAc3NeuAc1 + $1SO_4$ + 2Na	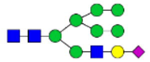	1	2	Hyb, Sia, Sul
2341.791	2341	Hex6HexNAc5NeuAc1 + 2Na	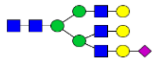	0	2	Tri, Sia
2361.677	2361 / 2362	Hex8HexNAc3NeuAc1 + $1SO_4$ + 3Na	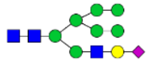	1	3	Hyb, Sia, Sul
2369.693	2369	Hex5HexNAc4NeuAc2 + $1SO_4$ + 3Na	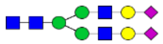	1	3	Bia, Sia, Sul
2377.851	2377	Hex6dHex1HexNAc6 + 1Na	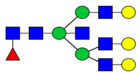	0	1	Fuc, Tet
2391.830	2391	Hex5dHex1HexNAc4NeuAc2 + 1Na	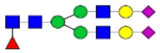	0	1	Fuc, Bia, Sia
2393.846	2393	Hex7HexNAc6 + 1Na	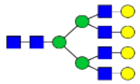	0	1	Tet

2413.812	2413	Hex5dHex1HexNAc4NeuAc2 + 2Na	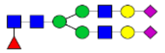	0	2	Fuc, Bia, Sia
2421.748	2422	Hex6HexNAc5NeuAc1 + 1SO_4 + 2Na	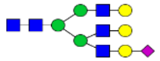	1	2	Tri, Sia, Sul
2435.794	2435	Hex5dHex1HexNAc4NeuAc2 + 3Na	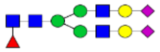	0	3	Fuc, Bia, Sia
2465.867	2465	Hex6dHex1HexNAc5NeuAc1 + 1Na	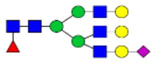	0	1	Fuc, Tri, Sia
2466.887	2466	Hex6dHex3HexNAc5 + 1Na	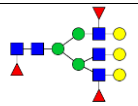	0	1	Fuc, Tri
2487.849	2487 / 2488	Hex6dHex1HexNAc5NeuAc1 + 2Na	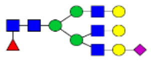	0	2	Fuc, Tri, Sia
2523.909	2523	Hex6dHex2HexNAc6 + 1Na	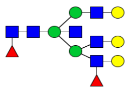	0	1	Fuc, Tet
2537.733	2537	Hex4dHex1HexNAc4NeuAc2 + 1SO_4 + 3Na	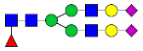	1	4	Fuc, Bia, Sia, Sul
2539.904	2539 / 2540	Hex7dHex1HexNAc6 + 1Na	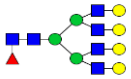	0	1	Fuc, Tet
2544.870	2544	Hex6HexNAc6NeuAc1 + 2Na	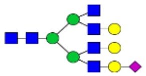	0	2	Tet, Sia
2550.790	2550	Hex5HexNAc5NeuAc2 + 1SO_4 + 2Na	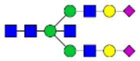	1	2	Bis, Sia, Sul
2594.894	2594	Hex5HexNAc5NeuAc2 + 1SO_4 + 4Na	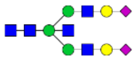	1	4	Bis, Sia, Sul
2610.904	2610	Hex6HexNAc5NeuAc2 + 1Na	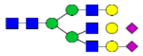	0	1	Tri, Sia

2616.892	2616	Hex5dHex1HexNAc5NeuAc2 + 2Na	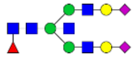	0	2	Fuc, Bis, Sia
2632.886	2632	Hex6HexNAc5NeuAc2 + 2Na	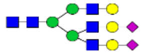	0	2	Tri, Sia
2633.907	2633	Hex6dHex2HexNAc5NeuAc1 + 2Na	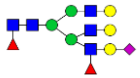	0	2	Fuc, Tri, Sia
2638.873	2638	Hex5dHex1HexNAc5NeuAc2 + 3Na	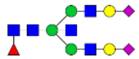	0	3	Fuc, Bis, Sia
2641.843	2642	Hex7dHex1HexNAc6 + 1SO_4 + 2Na	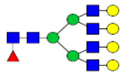	1	2	Fuc, Tet, Sul
2654.868	2654 / 2655	Hex6HexNAc5NeuAc2 + 3Na	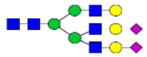	0	3	Tri, Sia
2669.967	2669	Hex6dHex3HexNAc6 + 1Na	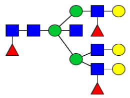	0	1	Fuc, Tet
2690.928	2691	Hex6dHex1HexNAc6NeuAc1 + 2Na	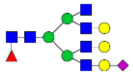	0	2	Fuc, Tet, Sia
2735.846	2735	Hex6dHex2HexNAc5NeuAc1 + 1SO_4 + 3Na	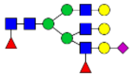	1	3	Fuc, Tri, Sia, Sul
2756.962	2756	Hex6dHex1HexNAc5NeuAc2 + 1Na	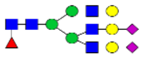	0	1	Fuc, Tri, Sia
2757.983	2757	Hex6dHex3HexNAc5NeuAc1 + 1Na	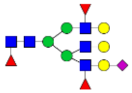	0	1	Fuc, Tri, Sia
2778.934	2778	Hex6dHex1HexNAc5NeuAc2 + 1Na	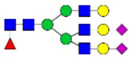	0	2	Fuc, Tri, Sia
2800.926	2800 / 2801	Hex6dHex1HexNAc5NeuAc2 + 3Na	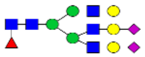	0	3	Fuc, Tri, Sia

2852.981	2852 / 2853	Hex7dHex1HexNAc6NeuAc1 + 2Na	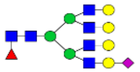	0	2	Fuc, Tet, Sia
2923.979	2923	Hex6dHex1HexNAc5NeuAc2 + 1SO4 + 4Na	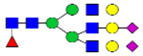	1	4	Fuc, Tri, Sia, Sul
2945.964	2945	Hex6HexNAc5NeuAc3 + 3Na	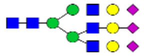	0	3	Tri, Sia
2967.946	2967 / 2968	Hex6HexNAc5NeuAc3 + 4Na	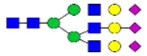	0	4	Tri, Sia
3004.005	3004	Hex6dHex1HexNAc6NeuAc2 + 3Na	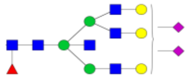	0	3	Bis, Fuc, Tri, Sia
3048.058	3048	Hex6dHex1HexNAc5NeuAc3 + 1Na	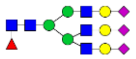	0	1	Fuc, Tri, Sia
3070.040	3070	Hex6dHex1HexNAc5NeuAc3 + 2Na	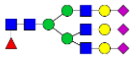	0	2	Fuc, Tri, Sia
3092.022	3092 / 3093	Hex6dHex1HexNAc5NeuAc3 + 3Na	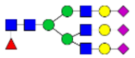	0	3	Fuc, Tri, Sia
3114.004	3113 / 3114	Hex6dHex1HexNAc5NeuAc3 + 4Na	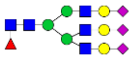	0	4	Fuc, Tri, Sia
3144.077	3144	Hex7dHex1HexNAc6NeuAc2 + 2Na	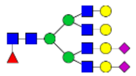	0	2	Fuc, Tet, Sia
3166.059	3166	Hex7dHex1HexNAc6NeuAc2 + 3Na	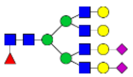	0	3	Fuc, Tet, Sia
3193.086	3193	Hex6dHex1HexNAc5NeuAc3 + 1SO4 + 3Na	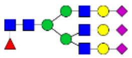	1	2	Fuc, Tri, Sia, Sul
3267.137	3267	Hex7HexNAc6NeuAc3 + 2Na	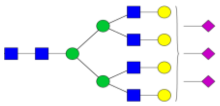	0	2	Tri, Sia

3311.106	3311	Hex7HexNAc6NeuAc3 + 3Na	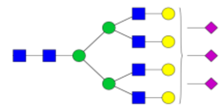	0	3	Tri, Sia
3333.074	3333	Hex7HexNAc6NeuAc3 + 4Na	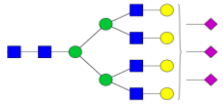	0	4	Tri, Sia
3384.116	3384	Hex8HexNAc8Fuc4 + 1Na	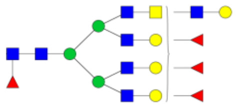	0	1	Fuc,Tet
3479.136	3479	Hex7dHex1HexNAc6NeuAc3 + 4Na	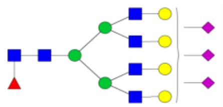	0	4	Fuc, Tet, Sia
3486.141	3486	Hex8HexNAc8Fuc4 + 1SO4 + 2Na	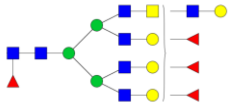	1	2	Fuc,Tet, Sul
3646.247	3646	Hex7HexNAc6NeuAc4 + 5Na	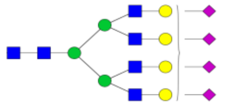	0	5	Tet, Sia
3770.314	3770	Hex7dHex1HexNAc6NeuAc4 + 4Na	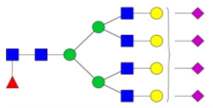	0	4	Fuc, Tet, Sia
3792.342	3792	Hex7dHex1HexNAc6NeuAc4 + 5Na	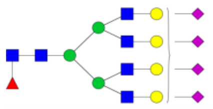	0	5	Fuc, Tet, Sia

Fuc = fucosylation, Sia = sialylation, Man = high-mannose, Hyb = hybrid, Bia = biantennary, Bis = bisecting, Tri = triantennary, Tet = tetraantennary, Sul = sulfation, Alp = alpha-galactose. The number of sulfates present is indicated in a separate column (+SO4). Glycosylation Class = class(es) each glycan was included for Fig. 2, Fig. 4, and Tables 2, 3, S2, S3.

Table S2. Mean difference and 95% confidence interval in the relative frequencies of the urine glycans.

Peak	LN vs. Healthy (95% CI)	Q-value	Male vs. Female (95% CI)	Q-value
1419	-1.054 (-1.331 ,-0.777)	3.88E-07	-0.321 (-0.598 ,-0.044)	0.2004
1853	-0.441 (-0.554 ,-0.328)	3.88E-07	0.003 (-0.11 ,0.116)	0.9770
2122	-2.637 (-3.323 ,-1.95)	3.88E-07	-0.714 (-1.4 ,-0.027)	0.2236
2361*	0.345 (0.258 ,0.432)	3.88E-07	0.28 (0.193 ,0.367)	4.00E-05
1831	-0.416 (-0.534 ,-0.298)	1.41E-06	-0.007 (-0.125 ,0.111)	0.9381
mannose	-2.796 (-3.609 ,-1.984)	1.93E-06	-0.977 (-1.79 ,-0.165)	0.2004
1581	-0.734 (-0.957 ,-0.51)	3.55E-06	-0.316 (-0.539 ,-0.092)	0.1395
2967	1.445 (1.006 ,1.884)	3.55E-06	0.289 (-0.15 ,0.728)	0.4887
2174	-0.452 (-0.596 ,-0.308)	7.57E-06	-0.166 (-0.31 ,-0.022)	0.2004
1257	-0.42 (-0.555 ,-0.285)	7.92E-06	-0.164 (-0.299 ,-0.029)	0.2004
2289*	5.116 (3.351 ,6.881)	2.21E-05	3.184 (1.419 ,4.949)	0.0439
2377	-0.606 (-0.814 ,-0.397)	2.21E-05	-0.236 (-0.444 ,-0.027)	0.2004
2012	-1.008 (-1.373 ,-0.644)	4.28E-05	-0.463 (-0.827 ,-0.098)	0.2004
2056	-0.235 (-0.321 ,-0.149)	4.69E-05	-0.044 (-0.13 ,0.042)	0.5766
2221	0.144 (0.091 ,0.198)	5.72E-05	0.07 (0.017 ,0.124)	0.1955
1866	-0.262 (-0.367 ,-0.158)	0.0001	-0.117 (-0.221 ,-0.012)	0.2033
tetraantennary	-1.494 (-2.074 ,-0.914)	0.0001	-0.249 (-0.829 ,0.331)	0.6218
1996	-0.109 (-0.152 ,-0.067)	0.0001	-0.009 (-0.052 ,0.033)	0.7953
2669	-0.367 (-0.509 ,-0.225)	0.0001	0.002 (-0.14 ,0.144)	0.9781
1891	-0.31 (-0.439 ,-0.181)	0.0002	-0.038 (-0.167 ,0.091)	0.7160
2158	-0.564 (-0.797 ,-0.331)	0.0002	-0.059 (-0.292 ,0.174)	0.7656
2852	-0.155 (-0.22 ,-0.089)	0.0002	-0.062 (-0.127 ,0.004)	0.2686
3333	0.175 (0.104 ,0.246)	0.0002	-0.026 (-0.097 ,0.045)	0.6787
1809	-1.532 (-2.191 ,-0.873)	0.0003	-0.684 (-1.343 ,-0.025)	0.2236
2267	1.34 (0.762 ,1.919)	0.0003	0.603 (0.025 ,1.182)	0.2236
2632	0.41 (0.233 ,0.588)	0.0003	-0.062 (-0.24 ,0.115)	0.6833
1743	-0.277 (-0.402 ,-0.153)	0.0005	-0.073 (-0.198 ,0.051)	0.5239
2100	-0.674 (-0.983 ,-0.365)	0.0005	-0.158 (-0.467 ,0.151)	0.5766
2245	0.795 (0.432 ,1.158)	0.0005	0.298 (-0.065 ,0.661)	0.3481
2487	-0.567 (-0.826 ,-0.309)	0.0005	-0.243 (-0.502 ,0.015)	0.2686
2945	0.3 (0.16 ,0.44)	0.0007	-0.008 (-0.148 ,0.132)	0.9381
sialylated	6.129 (3.082 ,9.176)	0.0011	3.448 (0.401 ,6.495)	0.2004
1485	2.221 (1.117 ,3.325)	0.0011	0.165 (-0.939 ,1.269)	0.8689
1704	-0.105 (-0.157 ,-0.054)	0.0011	-0.026 (-0.078 ,0.025)	0.5766
2465	-0.128 (-0.19 ,-0.066)	0.0011	-0.058 (-0.12 ,0.004)	0.2686
2523	-0.18 (-0.269 ,-0.091)	0.0011	-0.041 (-0.13 ,0.048)	0.6104
2654	0.822 (0.414 ,1.23)	0.0011	0.006 (-0.402 ,0.414)	0.9781
2287	0.378 (0.186 ,0.57)	0.0014	0.276 (0.084 ,0.468)	0.1395
2435	-1.108 (-1.668 ,-0.547)	0.0014	-0.076 (-0.637 ,0.484)	0.8724
1663	-0.799 (-1.214 ,-0.384)	0.0017	0.031 (-0.384 ,0.446)	0.9370
2383	0.035 (0.017 ,0.053)	0.0019	0.027 (0.009 ,0.045)	0.1395
1905	-0.31 (-0.482 ,-0.139)	0.0030	-0.1 (-0.272 ,0.071)	0.5239
3144	-0.045 (-0.07 ,-0.02)	0.0035	-0.021 (-0.046 ,0.004)	0.3481
2339*	0.264 (0.114 ,0.415)	0.0037	0.343 (0.193 ,0.494)	0.0046
2393	-0.135 (-0.211 ,-0.059)	0.0037	-0.053 (-0.129 ,0.023)	0.4677
2923	0.198 (0.085 ,0.311)	0.0038	-0.018 (-0.131 ,0.095)	0.8616
2610	0.223 (0.092 ,0.355)	0.0050	-0.026 (-0.157 ,0.106)	0.8317
triantennary	2.608 (1.056 ,4.16)	0.0053	-0.509 (-2.061 ,1.043)	0.6992
1960	0.082 (0.033 ,0.131)	0.0054	-0.006 (-0.055 ,0.043)	0.8852
fucosylated	-7.175 (-11.564 ,-2.786)	0.0062	-3.621 (-8.01 ,0.768)	0.3481
2304	-0.264 (-0.425 ,-0.103)	0.0062	-0.069 (-0.23 ,0.092)	0.6218
2319	0.099 (0.038 ,0.16)	0.0062	-0.039 (-0.1 ,0.022)	0.4924
1814	0.048 (0.018 ,0.079)	0.0080	0.014 (-0.016 ,0.045)	0.6063
2163	-0.334 (-0.546 ,-0.121)	0.0082	-0.122 (-0.334 ,0.091)	0.5290
3646	0.065 (0.023 ,0.107)	0.0093	-0.024 (-0.066 ,0.018)	0.5290
3770	-0.031 (-0.05 ,-0.011)	0.0094	-0.008 (-0.028 ,0.011)	0.6218
sulfated	-0.74 (-1.229 ,-0.251)	0.0105	0.51 (0.021 ,0.999)	0.2236
2413	-0.281 (-0.469 ,-0.092)	0.0118	-0.055 (-0.243 ,0.134)	0.7160
1444	-0.065 (-0.11 ,-0.021)	0.0125	-0.044 (-0.089 ,0)	0.2509
1850	-0.537 (-0.911 ,-0.164)	0.0144	-0.398 (-0.772 ,-0.025)	0.2236
2391	-0.089 (-0.152 ,-0.027)	0.0151	-0.018 (-0.081 ,0.044)	0.7160
3093	0.096 (0.028 ,0.164)	0.0156	0.008 (-0.06 ,0.076)	0.8852
3113	0.264 (0.077 ,0.45)	0.0156	0.156 (-0.031 ,0.342)	0.3481
1647	1.491 (0.424 ,2.557)	0.0165	-0.101 (-1.168 ,0.965)	0.9129
1954	0.568 (0.152 ,0.984)	0.0191	0.292 (-0.124 ,0.708)	0.4660
bisecting	-2.236 (-3.916 ,-0.557)	0.0216	-1.296 (-2.976 ,0.383)	0.3938
3092	0.064 (0.016 ,0.111)	0.0216	0.01 (-0.037 ,0.058)	0.7953
1079	-0.209 (-0.37 ,-0.048)	0.0252	-0.188 (-0.349 ,-0.027)	0.2004
2638	0.347 (0.078 ,0.616)	0.0255	-0.094 (-0.363 ,0.175)	0.6833
1611	0.063 (0.014 ,0.112)	0.0264	0.031 (-0.018 ,0.08)	0.5037
3004	-0.062 (-0.111 ,-0.013)	0.0275	-0.02 (-0.069 ,0.029)	0.6461
2028	-0.072 (-0.128 ,-0.015)	0.0287	-0.09 (-0.146 ,-0.033)	0.1114
1773	0.516 (0.094 ,0.937)	0.0323	-0.156 (-0.577 ,0.266)	0.6787
3384	-0.034 (-0.063 ,-0.006)	0.0323	-0.014 (-0.043 ,0.014)	0.5766
2594	0.024 (0.004 ,0.044)	0.0346	0.012 (-0.008 ,0.032)	0.5239
3193	-0.032 (-0.06 ,-0.005)	0.0346	-0.013 (-0.041 ,0.014)	0.5863
1875	-0.029 (-0.054 ,-0.005)	0.0353	-0.003 (-0.028 ,0.021)	0.8724
2341	0.214 (0.03 ,0.398)	0.0401	-0.061 (-0.245 ,0.123)	0.6992
1751	0.132 (0.012 ,0.252)	0.0526	-0.094 (-0.214 ,0.026)	0.3903
1837	-0.091 (-0.176 ,-0.005)	0.0625	0.062 (-0.023 ,0.148)	0.4360
1298	-0.026 (-0.052 ,-0.001)	0.0650	-0.004 (-0.03 ,0.021)	0.8502
1501	0.065 (0.001 ,0.129)	0.0736	0.016 (-0.048 ,0.08)	0.7656
2757	-0.041 (-0.087 ,0.004)	0.1056	-0.053 (-0.099 ,-0.008)	0.2004
1460	0.142 (-0.015 ,0.299)	0.1096	-0.098 (-0.255 ,0.059)	0.5037
2026	-0.037 (-0.078 ,0.005)	0.1182	0.02 (-0.021 ,0.062)	0.5863
3166	-0.074 (-0.161 ,0.012)	0.1271	-0.026 (-0.113 ,0.06)	0.7160
1757	-0.03 (-0.065 ,0.006)	0.1331	-0.021 (-0.056 ,0.015)	0.5239
2616	0.035 (-0.011 ,0.081)	0.1765	-0.035 (-0.081 ,0.011)	0.3981
1910	-0.092 (-0.224 ,0.04)	0.2190	0.048 (-0.084 ,0.18)	0.6787
2433	-0.024 (-0.066 ,0.017)	0.2992	-0.019 (-0.061 ,0.022)	0.6063
3792	-0.054 (-0.145 ,0.037)	0.2992	-0.015 (-0.106 ,0.076)	0.8616
1688	0.137 (-0.098 ,0.373)	0.3028	-0.088 (-0.323 ,0.148)	0.6787
2800	-0.181 (-0.513 ,0.151)	0.3371	-0.097 (-0.429 ,0.235)	0.7160
biantennary	1.154 (-1.402 ,3.71)	0.4321	3.045 (0.489 ,5.601)	0.2004
3479	-0.025 (-0.08 ,0.03)	0.4321	-0.018 (-0.073 ,0.037)	0.6992
1815	0.016 (-0.02 ,0.052)	0.4401	0.032 (-0.004 ,0.068)	0.3143
2544	-0.025 (-0.087 ,0.037)	0.4758	0.06 (-0.002 ,0.122)	0.2658
2303	-0.034 (-0.123 ,0.054)	0.4893	-0.058 (-0.146 ,0.031)	0.4887
3048	0.014 (-0.024 ,0.053)	0.5003	0.017 (-0.021 ,0.056)	0.6104
2325	-0.09 (-0.421 ,0.24)	0.6366	-0.128 (-0.459 ,0.202)	0.6707
3070	-0.005 (-0.026 ,0.015)	0.6420	-0.013 (-0.034 ,0.007)	0.4887
2778	-0.025 (-0.126 ,0.076)	0.6612	-0.044 (-0.145 ,0.057)	0.6218
1976	-0.147 (-0.977 ,0.683)	0.7589	0.46 (-0.37 ,1.29)	0.5445
2537	-0.006 (-0.042 ,0.03)	0.7693	-0.034 (-0.07 ,0.002)	0.2686
1825	0.008 (-0.057 ,0.073)	0.8251	-0.06 (-0.125 ,0.005)	0.2686
2756	0.001 (-0.026 ,0.027)	0.9799	0.018 (-0.009 ,0.044)	0.4887
hybrid	0.009 (-0.854 ,0.871)	0.9847	0.051 (-0.812 ,0.913)	0.9381

Mean difference and 95% confidence interval in the relative frequencies of the urine glycans or glycan groups between LN and HC and between females and males were calculated. Q-values reported are the Benjamini-Hochberg FDR corrected values for (1) the difference between HC and LN and (2) between males and females. Values in grey are for those peaks that were not significantly different between HC and LN after FDR correction. *N-glycans significantly different between females and males.

Table S3. Mean difference and 95% confidence interval in the relative frequencies of the serum glycans.

Peak	LN vs. Healthy (95% CI)	Q-value	Male vs. Female (95% CI)	Q-value	Peak	LN vs. Healthy (95% CI)	Q-value	Male vs. Female (95% CI)	Q-value
**high mannose**	-1.147 (-1.546 ,-0.749)	0.0002	-0.011 (-0.41 ,0.387)	0.9693	**2801**	0.07 (-0.016 ,0.157)	0.2310	0.033 (-0.053 ,0.12)	0.6634
**sulfated**	1.63 (1.052 ,2.207)	0.0002	0.298 (-0.28 ,0.875)	0.5584	**1688**	0.146 (-0.036 ,0.327)	0.2351	-0.093 (-0.275 ,0.088)	0.5584
**triantennary**	3.313 (2.028 ,4.598)	0.0004	-0.608 (-1.893 ,0.677)	0.5584	**1079**	-0.012 (-0.027 ,0.004)	0.2652	-0.009 (-0.024 ,0.007)	0.5584
**2275**	0.733 (0.429 ,1.036)	0.0007	-0.232 (-0.536 ,0.071)	0.4529	**1298**	0.143 (-0.052 ,0.338)	0.2851	0.26 (0.065 ,0.455)	0.4132
**1419**	-0.305 (-0.433 ,-0.177)	0.0007	-0.008 (-0.136 ,0.12)	0.9693	**2174**	0.254 (-0.094 ,0.602)	0.2851	0.225 (-0.123 ,0.573)	0.5243
**1743**	-0.224 (-0.319 ,-0.13)	0.0007	-0.04 (-0.135 ,0.054)	0.6066	**2466**	0.028 (-0.011 ,0.066)	0.2936	-0.013 (-0.052 ,0.025)	0.6886
**1905**	-0.318 (-0.454 ,-0.183)	0.0007	0.028 (-0.108 ,0.163)	0.8697	**hybrid**	-0.476 (-1.182 ,0.23)	0.3284	-0.016 (-0.722 ,0.69)	0.9693
**1809**	-2.168 (-3.099 ,-1.237)	0.0007	-0.586 (-1.517 ,0.345)	0.5252	**2422**	0.186 (-0.105 ,0.478)	0.3600	0.15 (-0.141 ,0.442)	0.5584
**1581**	-0.115 (-0.167 ,-0.064)	0.0011	-0.005 (-0.057 ,0.046)	0.9582	**2259**	0.133 (-0.081 ,0.346)	0.3745	-0.142 (-0.356 ,0.071)	0.5243
**2028**	0.858 (0.439 ,1.278)	0.0025	-0.318 (-0.738 ,0.101)	0.4529	**3114**	0.021 (-0.016 ,0.058)	0.4285	0.018 (-0.019 ,0.055)	0.5584
**2523**	0.218 (0.108 ,0.329)	0.0035	-0.1 (-0.211 ,0.01)	0.4473	**1501**	0.066 (-0.054 ,0.186)	0.4481	0.162 (0.042 ,0.282)	0.4132
**tetraantennary**	0.493 (0.235 ,0.751)	0.0045	-0.243 (-0.501 ,0.015)	0.4473	**1460**	-0.253 (-0.718 ,0.213)	0.4481	-0.012 (-0.477 ,0.454)	0.9693
**2540**	0.113 (0.051 ,0.176)	0.0073	-0.008 (-0.07 ,0.055)	0.9482	**2012**	-0.395 (-1.143 ,0.353)	0.4591	-0.264 (-1.012 ,0.484)	0.6886
**2968**	0.094 (0.041 ,0.146)	0.0076	-0.039 (-0.092 ,0.013)	0.4529	**2180**	0.016 (-0.015 ,0.046)	0.4762	-0.014 (-0.045 ,0.016)	0.5584
**1136**	0.111 (0.048 ,0.173)	0.0076	0.059 (-0.004 ,0.121)	0.4473	**2267**	0.056 (-0.055 ,0.166)	0.4762	0.098 (-0.013 ,0.208)	0.4473
**2633**	0.095 (0.041 ,0.15)	0.0076	-0.045 (-0.099 ,0.01)	0.4529	**2100**	-0.043 (-0.131 ,0.045)	0.4841	-0.007 (-0.095 ,0.081)	0.9693
**2319**	0.125 (0.054 ,0.197)	0.0079	-0.073 (-0.145 ,-0.002)	0.4473	**1825**	-0.072 (-0.23 ,0.085)	0.5191	-0.166 (-0.324 ,-0.009)	0.4473
**1444**	-0.211 (-0.334 ,-0.088)	0.0081	-0.055 (-0.178 ,0.068)	0.5854	**1850**	-0.111 (-0.358 ,0.137)	0.5290	-0.131 (-0.378 ,0.117)	0.5584
**2655**	0.234 (0.098 ,0.371)	0.0081	-0.093 (-0.229 ,0.044)	0.5243	**1606**	-0.023 (-0.074 ,0.029)	0.5315	-0.01 (-0.061 ,0.042)	0.8743
**2231**	-0.059 (-0.095 ,-0.023)	0.0102	-0.026 (-0.062 ,0.01)	0.4731	**2369**	0.014 (-0.019 ,0.047)	0.5355	-0.008 (-0.041 ,0.025)	0.8161
**2341**	0.426 (0.168 ,0.684)	0.0102	-0.225 (-0.483 ,0.033)	0.4473	**2037**	-0.007 (-0.026 ,0.011)	0.5579	-0.009 (-0.028 ,0.009)	0.5584
**1647**	-1.716 (-2.799 ,-0.632)	0.0141	-0.604 (-1.688 ,0.479)	0.5584	**933**	-0.015 (-0.051 ,0.022)	0.5670	-0.006 (-0.043 ,0.03)	0.8756
**2393**	0.208 (0.067 ,0.35)	0.0234	-0.118 (-0.26 ,0.023)	0.4529	**2550**	-0.011 (-0.039 ,0.017)	0.5670	0.015 (-0.013 ,0.043)	0.5584
**1257**	-0.101 (-0.173 ,-0.028)	0.0351	0.017 (-0.055 ,0.09)	0.8161	**1976**	0.247 (-0.426 ,0.92)	0.5865	0.731 (0.058 ,1.404)	0.4473
**2122**	-0.498 (-0.875 ,-0.121)	0.0449	0.053 (-0.324 ,0.43)	0.9252	**2413**	0.008 (-0.015 ,0.032)	0.5865	-0.027 (-0.051 ,-0.004)	0.4473
**biantennary**	-2.061 (-3.635 ,-0.486)	0.0458	1.46 (-0.114 ,3.035)	0.4473	**1704**	0.011 (-0.021 ,0.044)	0.5885	-0.016 (-0.049 ,0.016)	0.5584
**1095**	-0.083 (-0.152 ,-0.015)	0.0644	-0.001 (-0.07 ,0.067)	0.9693	**2435**	-0.028 (-0.117 ,0.06)	0.6283	-0.016 (-0.105 ,0.072)	0.8743
**2157**	0.12 (0.022 ,0.219)	0.0644	0.088 (-0.011 ,0.186)	0.4473	**2018**	-0.008 (-0.036 ,0.019)	0.6293	-0.02 (-0.048 ,0.007)	0.4529
**2303**	0.055 (0.009 ,0.102)	0.0721	-0.036 (-0.082 ,0.011)	0.4529	**2325**	-0.098 (-0.418 ,0.221)	0.6293	-0.161 (-0.481 ,0.158)	0.5584
**1910**	0.555 (0.084 ,1.026)	0.0721	0.544 (0.073 ,1.015)	0.4473	**1663**	0.386 (-0.908 ,1.681)	0.6346	1.135 (-0.16 ,2.429)	0.4473
**1954**	0.152 (0.003 ,0.302)	0.1339	0.124 (-0.025 ,0.274)	0.4529	**2289**	0.105 (-0.254 ,0.464)	0.6350	0.467 (0.108 ,0.826)	0.4132
**1339**	0.087 (0.001 ,0.173)	0.1339	0.026 (-0.06 ,0.112)	0.7405	**1282**	-0.027 (-0.124 ,0.07)	0.6479	-0.065 (-0.162 ,0.032)	0.5243
**sialylated**	1.661 (0.018 ,3.304)	0.1339	0.825 (-0.818 ,2.468)	0.5584	**2062**	0.005 (-0.016 ,0.026)	0.6979	-0.008 (-0.029 ,0.013)	0.6636
**fucosylated**	-2.425 (-4.909 ,0.058)	0.1495	-1.939 (-4.423 ,0.544)	0.4529	**1622**	-0.002 (-0.016 ,0.011)	0.7630	-0.006 (-0.02 ,0.007)	0.5584
**1485**	0.829 (-0.041 ,1.7)	0.1594	-0.459 (-1.33 ,0.411)	0.5584	**2245**	0.013 (-0.061 ,0.087)	0.7630	0.022 (-0.052 ,0.096)	0.7405
**1866**	0.067 (-0.006 ,0.141)	0.1745	-0.024 (-0.098 ,0.049)	0.7029	**2056**	-0.037 (-0.252 ,0.177)	0.7630	0.015 (-0.2 ,0.229)	0.9693
**2735**	0.046 (-0.005 ,0.097)	0.1745	0.001 (-0.05 ,0.052)	0.9693	**1611**	-0.003 (-0.024 ,0.018)	0.8019	0.021 (0 ,0.042)	0.4473
**1773**	-0.197 (-0.415 ,0.02)	0.1745	0.013 (-0.205 ,0.23)	0.9693	**2537**	-0.003 (-0.033 ,0.027)	0.8552	-0.019 (-0.049 ,0.011)	0.5243
**2488**	0.144 (-0.022 ,0.309)	0.1984	0.004 (-0.161 ,0.17)	0.9693	**bisecting**	-0.084 (-1.556 ,1.388)	0.9116	-0.767 (-2.239 ,0.705)	0.5584
**1960**	-0.023 (-0.052 ,0.005)	0.2300	0.019 (-0.01 ,0.047)	0.5243					

Mean difference and 95% confidence interval in the relative frequencies of the serum glycans or glycan groups between LN and HC and between females and males were calculated. Q-values reported are the Benjamini-Hochberg FDR corrected values for (1) the difference between HC and LN and (2) between males and females. Values in grey are for those peaks that were not significantly different between HC and LN after FDR correction. None of the glycans were significantly different between females and males.
